# CRISPR screens with trastuzumab emtansine in HER2-positive breast cancer cell lines reveal new insights into drug resistance

**DOI:** 10.1186/s13058-025-02000-1

**Published:** 2025-03-31

**Authors:** Barbara A. Lipert, Kyla N. Siemens, Aziza Khan, Rebecca Airey, Gech Heng Dam, Man Lu, Marcella Flinterman, Queenie Yong, Tet Woo Lee, Francis W. Hunter, Stephen M. F. Jamieson

**Affiliations:** 1 Auckland Cancer Society Research Centre, University of Auckland, Auckland, New Zealand; 2 Maurice Wilkins Centre for Molecular Biodiscovery, University of Auckland, Auckland, New Zealand; 3 Oncology Therapeutic Area, Johnson and Johnson Innovative Medicine, Spring House, PA USA; 4 Department of Pharmacology and Clinical Pharmacology, University of Auckland, Auckland, New Zealand

**Keywords:** T-DM1, HER2-positive breast cancer, Antibody-drug conjugates, CRISPR/Cas9, Functional genomics, Drug resistance, TSC1, TSC2

## Abstract

**Background:**

Trastuzumab emtansine (T-DM1) is an antibody–drug conjugate that is an effective therapy for HER2-positive breast cancer; however, its efficacy is limited by drug resistance. While multiple mechanisms of resistance have been proposed, these are not yet well understood. Greater understanding of T-DM1 sensitivity and resistance could provide new combination strategies to overcome resistance or predictive biomarkers to guide therapy.

**Methods:**

We have conducted CRISPR/Cas9 functional genomics modifier screens in HER2-positive breast cancer cell lines to allow for unbiased discovery of T-DM1 sensitivity and resistance genes. Whole-genome knockout screens were carried out in MDA-MB-361 and MDA-MB-453 cells treated with T-DM1 and its payload cytotoxin DM1. Hits were validated in secondary T-DM1 screens using a focused single-guide RNA (sgRNA) library and subsequently by individual gene knockout.

**Results:**

The whole-genome CRISPR screens with T-DM1 and DM1 identified 599 genes as potential modifiers of T-DM1 sensitivity and resistance. Of these, 17 genes were significantly enriched and 3 genes depleted at *P* < 0.001 in either or both MDA-MB-361 and MDA-MB-453 libraries in the secondary screens. Among the top hits, were known T-DM1 sensitivity genes *ERBB2* and *SLC46A3*, in addition to negative regulators of mTOR complex 1: *TSC1* and *TSC2*. MDA-MB-453 clones with knockout of *TSC1* or partial knockout of *TSC2* were more resistant to T-DM1 than wild type cells in competition growth assays and to T-DM1 and other HER2 targeting therapies (T-DXd, lapatinib and neratinib) in growth inhibition assays, and had increased internalisation of T-DM1 at 6 h. T-DM1 and the mTOR inhibitor everolimus demonstrated synergistic activity at inhibiting cell proliferation at multiple T-DM1 concentrations across four HER2-positive breast cancer cell lines.

**Conclusions:**

Our CRISPR screening approach with T-DM1 in HER2-positive breast cancer cell lines identified genes not previously implicated in T-DM1 sensitivity or resistance, including *TSC1* and *TSC2*. These genes may inform new strategies to enhance T-DM1 therapy in the clinic.

**Supplementary Information:**

The online version contains supplementary material available at 10.1186/s13058-025-02000-1.

## Background

Elevated expression of transmembrane tyrosine kinase receptor HER2 (Human Epidermal growth factor Receptor 2) on the surface of tumour cells characterises ~ 15–20% of breast cancer presentations and is associated with an invasive subtype and poor prognosis [1, 2]. HER2 overexpression is driven by amplification of the *ERBB2* gene located at the long arm of human chromosome 17 (17q12) The humanised monoclonal antibody trastuzumab is a component of standard of care for multiple different treatment regimens in HER2-positive breast cancer [3–7]. Trastuzumab selectively targets the extracellular domain IV of the HER2 receptor and prevents its homo-dimerisation, thereby blockading the activation of downstream oncogenic signalling pathways (RAS/RAF/MEK/ERK and PI3K/AKT) that promote cell proliferation, survival and tumourigenesis [8–10]. However, in the metastatic setting, not all patients respond to trastuzumab, while those who do respond invariably develop resistance resulting in disease progression [3, 11, 12]. To counter this, additional HER2-targeting agents have been developed, including the antibody–drug conjugate (ADC) trastuzumab emtansine (T-DM1).

T-DM1 comprises trastuzumab covalently bound to a highly potent cytotoxin (DM1) via a non-cleavable MCC thioether linker [13]. Conjugation to trastuzumab promotes selective release of the DM1 cytotoxin in cancer cells overexpressing HER2. Upon binding to HER2, T-DM1 is internalised by receptor-mediated endocytosis, trastuzumab undergoes proteolysis and DM1 is transported from the lysosome into the cytoplasm where it binds to microtubules, inducing their depolymerisation and causing cell death [14]. T-DM1 also retains the mechanisms of action of trastuzumab, including blockade of HER2 signalling, inhibition of HER2 ectodomain shedding and antibody-dependent cell-mediated cytotoxicity [15]. T-DM1 is approved for the second-line treatment of HER2-positive metastatic breast cancer in patients previously treated with trastuzumab and a taxane [16, 17] and in the non-metastatic setting as an adjuvant treatment in women with residual disease following neoadjuvant trastuzumab and chemotherapy [18, 19]. Although treatment with T-DM1 significantly improves outcomes in these patient populations, just like all other HER2-targeting agents [20], therapeutic resistance remains a major impediment, with over 50% of patients in the second-line metastatic setting initially refractory to T-DM1, while acquired resistance typically develops in patients who initially respond [16, 17].

Understanding the resistance mechanisms of T-DM1 will allow identification of strategies to overcome T-DM1 resistance. Various potential resistance mechanisms to T-DM1 have been investigated, including those related to the subversion of trastuzumab-mediated effects. However, the most strongly supported mechanisms appear to be trastuzumab-independent and relate to the intracellular trafficking and metabolism of T-DM1 and impairment of DM1-mediated cytotoxicity [21]. Proposed resistance mechanisms supported at least in part by clinical or preclinical evidence include: (a) reduction in HER2 expression [22, 23], (b) HER2 expression heterogeneity [24, 25]; (c) reduction in HER2 binding [26]; (d) dysregulated PI3K signalling [27]; (e) signalling through alternative receptor tyrosine kinases [28]; (f) modulation of immune responses [29]; (g) altered internalisation [30, 31]; (h) endosomal transit [26] or lysosomal catabolism of HER2-TDM1 complexes [32, 33]; (i) reduced expression of the lysosomal transporter SLC46A3 [34–36];(j) increased expression of drug efflux transporters [22, 34, 37]; and, (k) escape from mitotic catastrophe and apoptosis [38, 39]. However, most of these mechanisms are poorly understood and certainly not observed in all cases of T-DM1 resistance. A greater understanding of T-DM1 resistance mechanisms is essential for improving therapeutic outcomes not only with this agent [21], but also for other HER2-targeting agents. For instance, any ADC processing-related or trastuzumab-dependent mechanisms of resistance may also promote resistance to the second-generation ADC trastuzumab deruxtecan (T-DXd), which is used in the treatment of HER2-positive breast, gastric and other solid tumours, HER2-low breast cancer and HER2-mutant non-small cell lung cancer [40–44].

Here, we have conducted CRISPR/Cas9 functional genomics modifier screens to allow for unbiased discovery of T-DM1 sensitivity and resistance genes. We carried out whole-genome knockout screens in cell lines confirmed to be HER2-positive and sensitive to T-DM1, but insensitive to trastuzumab, and validated our hits through a secondary screen using a focused single-guide RNA (sgRNA) library, followed by individual gene knockout.

## Methods

### Drugs

T-DM1 was provided by Genentech (South San Francisco, CA), as lyophilised powder. It was solubilised in sterile water, filter-sterilised and stored at 4 °C for up to one month. Trastuzumab was provided as overage from formulated (bacteriostatic water for injection) Herceptin vials by Auckland City Hospital pharmacy, filter sterilised, stored at 4 °C and refreshed monthly. T-DXd was provided by Daiichi-Sankyo (Tokyo, Japan) in 25 mM histidine buffer in 9% sucrose, pH 5.5. DM1 (as a free thiol). Lapatinib and neratinib were acquired from AK Scientific, Inc. (Union City, CA) and stored as single-use DMSO solutions at − 80 °C.

### Cells and culture

MCF7, MDA-MB-453, MDA-MB-361, HCC1954, ZR-75–1, BT-474, and SK-BR-3 cell lines were obtained from the American Type Culture Collection (ATCC; Manassas, VA). The cells were cultured at 37 °C in humidified incubators with atmospheric O_2_ and 5% CO_2_. The cell lines were continuously passaged for less than two months from cryopreserved, authenticated vials confirmed to be free of mycoplasma contamination (PlasmoTest Mycoplasma Detection Kit; InvivoGen, San Diego, CA). Cell line authentication was performed by DNA Diagnostics (Auckland, New Zealand) using short tandem repeat profiling. MCF7, MDA-MB-453, and MDA-MB-361 were maintained in DMEM/F12 medium, while HCC1954, ZR-75–1, and BT-474 were cultured in RPMI-1640 and SK-BR-3 was maintained in McCoy’s 5A medium (all Thermo Fisher Scientific, Auckland, New Zealand). All base media were supplemented with 10% heat-inactivated foetal calf serum (FCS; Moregate Biotech, Hamilton, New Zealand).

### Fluorescence in situ hybridisation (FISH)

FISH testing was performed by IGENZ (Auckland, New Zealand). Cells in logarithmic phase growth were treated with 0.5 mg/mL colcemid (Thermo Fisher Scientific) for 4–6 h, trypsinised, then resuspended in hypotonic solution 0.075 M KCl; Thermo Fisher Scientific) and incubated for 10 min at 37 °C. The cells were then fixed in Carnoy’s fixative (methanol-glacial acetic acid 3:1) and one drop of fixed cells added onto glass slides (Globe Scientific; Paramus, NJ). The slides were air-dried then dehydrated in 70% ethanol for 2 min, 80% ethanol for 2 min, and 100% ethanol for 2 min. After air-drying, CymoGenDx ERBB2 (17q12) + Copy Control 17 Green FISH Probe (Biocare Medical; Pacheco, CA) was added to the hybridisation site on each slide. Hybridisation sites were covered with cover slips (Thermo Fisher Scientific) and sealed with rubber cement. Slides were co-denatured at 73 °C for 5 min in a thermocycler and subsequently placed in a humidity chamber at 37 °C for 4 h. After incubation, cover slips were removed and slides were placed in a pre-warmed (74 °C) wash solution containing 0.4 × sodium saline citrate buffer for 2 min. Slides were then transferred to Coplin jars containing PBS for 5 min at 20 °C. Slides were then air-dried and 8 μL VECTASHIELD antifade (Abacus ALS; Queensland, Australia) was added to the hybridisation site on each slide. One slide per cell line was visualised and 100 cells were scored for ERBB2–CEP17 copy number ratio.

### Western blotting

Cells were detached from the surface using trypsin/EDTA solution, centrifuged at 90 × g for 5 min, and washed in PBS. The cell pellet was resuspended in ice-cold radio-immunoprecipitation assay (RIPA) buffer (150 mM NaCl, 1.0% IGEPAL® CA-630, 0.5% sodium deoxycholate, 0.1% SDS, 50 mM Tris, pH 8.0) supplemented with 1 × protease inhibitor cocktail and 0.5 mM sodium orthovanadate (phosphatase inhibitor; all Merck, Auckland, New Zealand), and incubated on ice for 10 min. The lysate was cleared by centrifugation at 10,000 × g for 10 min at 4 °C; the supernatant was stored at − 20 °C. The bicinchoninic acid assay (Thermo Fisher Scientific) was used to quantify the protein concentration in the lysates. For SDS-PAGE, proteins were denatured by mixing with 1 × Bolt LDS sample buffer (Thermo Fisher Scientific) containing 5% β-mercaptoethanol (Merck) at 1:4 ratio and incubated at 70 ℃ for 10 min. Electrophoresis was carried out using Bolt 4–12% Bis–Tris gradient gels (Thermo Fisher Scientific) in 1 × Bolt MES SDS running buffer (Thermo Fisher Scientific), loading 20 μg of total protein per lane alongside Precision Plus Protein Kaleidoscope protein marker (Bio-Rad Laboratories, Auckland, New Zealand). The Electrophoretic Transfer Cell System (Bio-Rad Laboratories) was used to transfer proteins to Immobilon-P 0.45 μm PVDF transfer membrane (Bio-Rad Laboratories), with wet transfer carried out at 100 V for 1 h. Membranes were cut using the protein marker as a guide to allow antibodies against proteins of different molecular weights to be tested on the same samples. Before immunoblotting, membranes were incubated in blocking buffer (5% BSA; MP Biochemicals, Auckland, New Zealand; in TBS-0.1% Tween) for 1 h, then incubated in primary antibody solution overnight in 5% BSA in TBS-0.1% Tween. Antibodies used included: anti-ERBB2 (clone 3B5, #SC-33684, Santa Cruz Biotechnology, dilution 1:1000), anti-TSC1 (clone 5C8A12, #37–0400, Thermo Fisher Scientific, dilution 1:2000), anti-TSC2 (clone D93F12, #4308 T, Cell Signaling Technology, Danvers, MA, dilution 1:2000), and anti-β-actin (clone C4, #mab1501, Merck, 0.5 μg/mL, dilution 1:10,000). After washing in TBS-0.1% Tween, membranes were incubated in anti-rabbit (#65–6120, Thermo Fisher Scientific, dilution 1:5000), or anti-mouse IgG-HRP (#62–6520, Thermo Fisher Scientific, dilution 1:5000) secondary antibody for 1 h at 20 °C with gentle agitation. The signal was detected using Pierce ECL SuperSignal West Pico substrate (Thermo Fisher Scientific).

### DNA content analysis

Single cells harvested from monolayers in log-phase growth were fixed in 70% ethanol, washed three times in PBS containing 3% FCS, filtered through a 40 µm strainer cap (Corning, New York, NY) then stained with 20 µg/mL propidium iodide (Merck) in the presence of 0.1 mg/mL RNAse (Roche, Auckland, New Zealand) at 20 °C in darkness for 10 min. Fluorescence was then measured using an Accuri C6 flow cytometer (BD Biosciences, San Jose, CA) with a 488 nm excitation laser and an FL3 (670 long pass) filter for emission. DNA content was assessed in ModFit LT (version 4.1.7, Verity Software House, Topsham, ME) and G1 peaks were used to assess ploidy relative to HCT116 cells included in experiments as an authentic diploid reference.

### Lentivirus production and MOI determination for whole genome screens

LentiCas9-Blast plasmid (#52962, Addgene, Watertown, MA) was used for Cas9 expression. The GeCKOv2 library was purchased as a two-vector system (#1000000049, Addgene) as a bacterial stab (lentiCas9-Blast) and lyophilised DNA preparations (GeCKOv2 half-libraries A and B; lentiGuide-Puro vector). The focused library was custom-designed using the lentiGuide-Puro vector backbone by Princess Margaret Genomics Centre (Toronto, Canada). The libraries were transformed into Lucigen Endura competent Escherichia coli (LGC Biosearch Technologies, Hoddesdon, UK) using Gene Pulsar Xcell™ electroporation system (Bio-Rad Laboratories) and amplified according to methods described by the Feng Zhang laboratory (http://sanjanalab.org/lib.html). Once amplified, GeCKOv2 library A and B were combined at equimolar concentrations.

All lentiviral plasmids were packaged in HEK293FT cells (Thermo Fisher Scientific) using the pMD2.G (#12259, Addgene) and psPAX2 (#12260, Addgene) packaging plasmids. Transfection was carried out using Lipofectamine 3000 (Thermo Fisher Scientific). Exactly 6 h post-transfection, the medium was replaced by fresh culture medium and lentiviral supernatant was harvested 24 h and 48 h post-transfection. LentiCas9-Blast lentiviral preparations were filtered and used unconcentrated. The combined GeCKOv2 lentiviral library and lentiviral preparations of the focused library were concentrated by centrifugation at 10,000 × g for 5 h at 4 °C with a 20% sucrose cushion. Lentivirus preparations were snap-frozen in liquid nitrogen and stored at − 80 °C until transduction.

To determine lentiviral dilutions for library infections at scale, viral stocks were titrated against each cell line. Cell lines were seeded at varying densities (depending on cell size and doubling time) in 6-well plates and incubated overnight. A dilution series of lentiviral preparations were added to the plates the following day. Media was supplemented with 8 μg/mL polybrene (Merck) to enhance transduction. The cells were incubated for 24 h, after which media was removed, and fresh media containing selective antibiotics at a predefined concentration was added to the cells to select for stable integration of the vector. Selection continued until the cells with no exposure to the virus but exposed to antibiotics were completely sterilised. The number of surviving cells was compared to a non-transduced control sample maintained in logarithmic growth.

### Pooled CRISPR depletion screens

The cells were plated in T175 flasks, and library transductions were carried out to achieve an initial coverage (i.e., transduced cells per sgRNA sequence) of at least 300–500-fold upon infection with GeCKOv2 library and 1000-fold upon infection with the focused library at an MOI of 0.1–0.3. After completing the antibiotic selection, surviving cells were harvested and seeded into T-175 flasks for screens with T-DM1, DM1 or no treatment. During the screens, cells were maintained and regularly passaged in 2–3 independent replicates for each condition, with the secondary screens repeated using duplicate transductions. A 300–500-fold library coverage was maintained throughout the screen for each replicate of the GeCKOv2 library, and a 1000-fold library coverage for each replicate of the focused library. Genomic DNA was isolated from the final passage and from pre-screened cultures (time zero samples) for sequencing.

### Genomic DNA extraction, library preparation, and sequencing

Genomic DNA was isolated from frozen cell pellets containing 20–50 × 10^6^ cells (GeCKOv2 library) or 5–10 × 10^6^ cells (focused library) using the QIAamp DNA Blood and Tissue Maxi Kit (Qiagen, Auckland, New Zealand) and purified by ethanol precipitation. Genomic DNA was then quantified using Qubit dsDNA BR Assay kit (Thermo Fisher Scientific) on a Qubit 3.0 fluorometer (Thermo Fisher Scientific) and purity was measured by spectrophotometry using a NanoDrop OneC Microvolume UV–Vis Spectrophotometer (Thermo Fisher Scientific). The sgRNA spanning region was amplified from purified genomic DNA. To this end, three separate polymerase chain reactions (PCRs) were performed on each sample. The first PCR (PCR1) amplified a region encompassing the 20 bp sgRNA, thus isolating it from host cell gDNA. The second PCR (PCR2) utilised primers with 3’ overhangs to add the barcodes, variable length staggers, and adapters required for Illumina sequencing. The final PCR (PCR3) used primers annealing to Illumina p7 and p5 sites to amplify the sequencing library. All PCRs were performed using Herculase II Fusion Enzyme, 4% DMSO, 100 mM dNTP mixture (all from Agilent Technologies, Auckland, New Zealand) and sterile water. All primers were HPLC-purified, while primers in PCR2 were Ultramer DNA oligos (Integrated DNA Technologies, Coralville, IA). PCR reactions were prepared on ice in a PCR Workstation (Hoefer, Holliston, MA) hood using sterile pipettes, filter tips, and autoclaved tubes. Each experiment included plasmid DNA as a positive control and sterile water as a negative control. All reactions were performed using an Eppendorf Nexus GX2 thermocycler (Hamburg, Germany).

### Analysis of CRISPR screens

Illumina FASTQ files for each sequenced library were demultiplexed and concatenated and their quality checked using FastQC. Demultiplexed reads were run through Cutadapt [45] to trim PCR and vector backbone adapter sequences flanking the sgRNA. Reads in which the trimmed adapters could not be identified without mismatches, or that contained base call failures, or that were not exactly 20 bp in length were discarded. Trimmed reads were aligned to the sgRNA sequence present in the respective sgRNA libraries using Burrows-Wheeler Aligner alignment [46], with the number of reads aligned to each sgRNA sequence in the library determined using SAMtools [47]. For the whole genome screens, gene level scores for enrichment or depletion of sgRNAs in drug-treated relative to control GeCKOv2 libraries were computed using MAGeCK [48], followed by aggregation of sgRNAs into genes using modified robust ranking aggregation. For the focused library screens, library sizes were normalised with the relative log expression (RLE) method and sgRNA-level analysis was performed using voom/limma [49] with the whole dataset fit to a single linear model blocked by cell line and screen repeat. Gene-level results were obtained by applying ROAST [50] to the set of sgRNAs targeting each gene; 9,999,999 rotations were used. For evaluation of essential genes, a list of essential genes was compiled from previous publications [51, 52].

#### Cas9 ribonucleoprotein transfection

Ribonucleoprotein (RNP) complexes consisted of gRNA and Cas9 nuclease 2NLS*, S. pyogenes* (Synthego CRISPR Gene Knockout Kit v2. Redwood City, CA). We utilised a mix of three gene-targeting crRNAs (*TSC1* protospacers: UCAUGGUGCCUGAAGAAACC, UUCCCAGACUGUGGAAUCAU, GACGUCGUUGUCCUCACAAC; *TSC2* protospacers: UCUUUAGGGCGAGCGUUUGG, CGUGAAGGUCUUCGUUGGAA, UGGGAGACACAUCACCUACU), each linked to Synthego modified EZ Scaffold). Cells were subcultured two days before nucleofection and collected at 80–90% confluency. For each nucleofection reaction, 4 × 10^5^ cells were aliquoted in microcentrifuge tubes and spun at 90 × g for 10 min. The cell pellet was washed with PBS and resuspended in 20 μL of SF Nucleofector™ solution mixed with supplement (Lonza, Basel, Switzerland). RNP complexes were formed according to Synthego’s protocol by mixing 120 pmol gRNA (total) and 20 pmol Cas9 and nucleofected using program DK-100 at 4D-Nucleofector™ X Unit (Lonza). After the nucleofection reaction, cells were resuspended using 80 μL of growth medium and split into two replicate wells containing 100 μL medium on a 96-well plate. The plates were incubated at 37 °C in a 5% CO_2_ incubator until single-cell dilution was carried out.

#### Antiproliferative assays

The half-maximal inhibitory concentrations (IC_50_) of T-DM1 and DM1 were determined in Cas9-expressing cells to identify suitable starting concentrations for the CRISPR screens using a 6-day proliferation assay with continuous drug exposure and a CellTiter-Glo viability endpoint. Cells were seeded at 37 °C with 5% CO_2_ overnight then treated with drugs in duplicate threefold dilution series. After 6 days’ exposure, CellTiter-Glo reagent (Promega, Madison, WI) was added at 1:4 dilution. Plates were mixed on an orbital plate shaker (125 rpm) for 2 min at 20 °C and then incubated without shaking at 20 °C for 10 min prior to measuring luminescence on an EnSpire Multimode Plate Reader (PerkinElmer; Waltham, MA). Antiproliferative activity of T-DM1, DM1, T-DXd, lapatinib and neratinib on MDA-MB-453 wild type and knockout cell lines (seeded at 4000 cells/well) after 5 days’ exposure was evaluated by sulforhodamine B assay as described previously [53]. Antiproliferative activity of T-DM1 in combination with everolimus was evaluated by thymidine incorporation assay. Cells were seeded for 6–8 h at 37 °C with 5% CO_2_ then treated with continuous exposure to T-DM1 in a threefold dilution series in duplicate and/or five different concentrations of everolimus for 3 days. ^3^H-thymidine (0.04 µCi per well, Perkin Elmer), was added to the cells for 5 h prior to harvest. The cells were harvested onto glass fibre filtermats (Perkin Elmer) using an automated TomTec Harvester 96 (Hamden, CT). Filtermats were left to dry then incubated with BetaPlate Scint (Perkin Elmer) and thymidine incorporation was measured in a Trilux/Betaplate counter (Perkin Elmer). For all antiproliferative assays, values were adjusted by subtraction of blanks without cells and normalised to untreated control cultures on the same plate to define concentration–response curves. Four-parameter nonlinear regressions were fitted to the data using Prism (Version 9.3.1; GraphPad Software, Inc., La Jolla, CA) to define IC_50_ values. Combination indices were calculated using CompuSyn v1.0 (ComboSyn Inc., Paramus, NJ) and synergy evaluated using previously reported definitions [54].

#### Clonogenic survival assay

The sensitivity of cell lines to trastuzumab was assessed using clonogenic survival assays. Cas9-expressing cell lines were seeded in 12 well plates at densities that varied according to cell size and doubling times and were incubated overnight. The next day, trastuzumab was added to a top well to a final concentration of 500 µM and serially diluted 1:3 over nine increments. The plates were then incubated for varying durations that differed between cell lines to provide optimal colony size and morphology. On the final day of drug exposure, the medium was removed, and colonies were stained by adding 0.5 mL methylene blue (Merck, 2 g/L in 50% ethanol) to each well and were incubated for 1 h at room temperature. The methylene blue was removed and the plates were dried. The following day, plates were imaged for qualitative (visual) assessment of inhibition of clonogenicity as a function of trastuzumab concentration. The clonogenic survival assay was modified slightly to assess T-DM1 sensitivity at the endpoint of CRISPR/Cas9 discovery screens. The top concentration was 1 µM diluted 1:3 over nine increments. The plates were incubated for 21 days before staining using the above-mentioned procedure.

#### Competition growth assay

Log-phase growing *TSC1* knockout MDA-MB-453 clones and polyclonal populations of *TSC1* wild-type MDA-MB-453 cells stably expressing humanised recombinant GFP under EF1α constitutive promoter (F527-hrGFP plasmid supplied by Dr Shevan Silva, University of Auckland) were trypsinised, washed in PBS and counted. Each knockout clone was mixed with GFP-positive cells at 1:1 ratio, and the combined populations were seeded at 4,000, 80,000 and 160,000 cells/well in 12-well plates overnight at 37 °C in a 5% CO_2_ incubator. The next day, T-DM1 was added to the cells at different concentrations and incubated for 5 days. Cells were then detached using trypsin/EDTA (Thermo Fisher Scientific) and plated onto new 12-well plates with fresh media containing T-DM1 and incubated for a further five days. After 5 days, the cells were collected from the plates, washed with PBS and the GFP signal in cell suspensions was measured using an Accuri C6 flow cytometer. The percentages of cells positive for GFP after treatment were compared to control cells by repeated measures one-way ANOVA with Dunnett’s multiple comparison test.

#### T-DM1 internalisation

T-DM1 was conjugated to Alexa Fluor 488 using Alexa Fluor 488 5-SDP Ester (ThermoFisher Scientific) and was separated from unconjugated drug on a Bio-Spin P-6 gel column (Bio-Rad). Cells were seeded at 1.2 × 10^5^ cells per well in an 8-well µ-slide (ibidi, Gräfelfing, Germany) and left for 24 h in a 5% CO_2_ incubator. Cells were washed in PBS and treated with 1 μM CellTrace Violet in PBS for 20 min, which was then removed and replaced by 75 nM LysoTracker Deep Red diluted in media with 1% FCS for 30 min. After another wash in PBS, cells were incubated with 40 µg/ml T-DM1 conjugated to Alexa Fluor 488 diluted in media with 1% FCS on ice in the dark for 1 h. Cells were washed twice with media with 1% FCS and then imaged using a LSM 710 inverted confocal microscope and a LSM 800 Airyscan confocal microscope (Zeiss, Jena, Germany). Alexa Fluor 488 intensity was measured in the cytoplasmic regions of multiple cells at 0, 1 and 6 h using Zen 2.5 and Zen 2.6 Software (Zeiss).

## Results

### Whole-genome CRISPR screens with T-DM1 and DM1

**Fig. 1:**
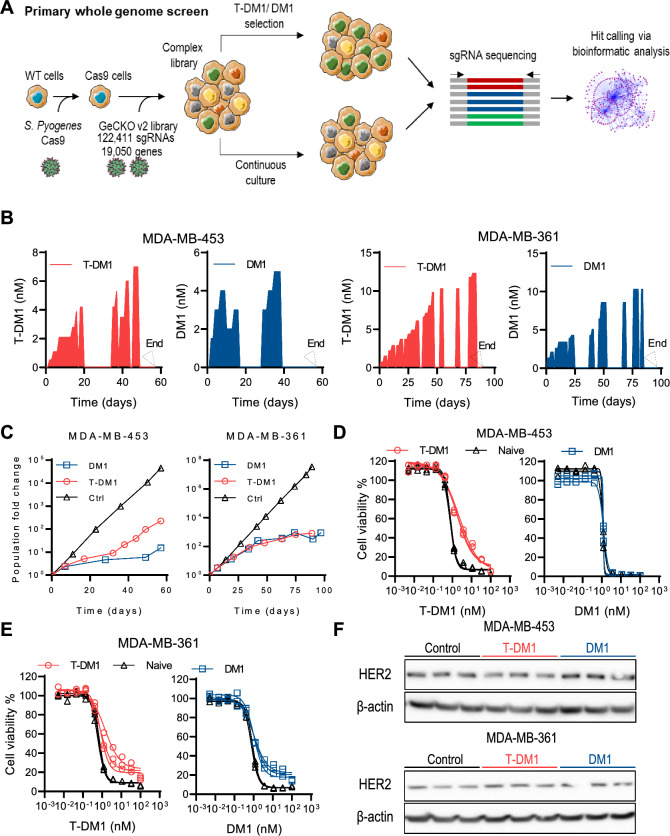
Whole-genome CRISPR/Cas9 knockout screens with T-DM1 and DM1 in MDA-MB-453 and MDA-MB-361 cells. **A** Schematic of whole-genome screens. **B** Concentration and schedule of T-DM1 and DM1 treatment during the screens. Arrows denote the time at which the screens were terminated. **C** Cell growth for the control (Ctrl) and drug-treated cultures during the screens (mean ± SEM, n = 3). **D** Emergence of resistance to T-DM1 and/or DM1 at the end of the screens in cultures that were treated with T-DM1 or DM1 during the screens relative to untreated (naïve) cultures in MDA-MB-453 and **E** MDA-MB-361 cells (n = 3). Cultures at the end of the screens were treated with DM1 or T-DM1 continuously for 6 days. **F** HER2 protein expression in control and drug-treated cultures at the end of the screen. β-actin was used as a protein loading control

Whole genome CRISPR-Cas9 screens were carried out in two HER2-positive breast cancer cell lines (MDA-MB-361 and MDA-MB-453) treated with T-DM1 and DM1 to identify genes involved in sensitivity and resistance to these agents (Fig. 1A). MDA-MB-361 and MDA-MB-453 were selected for screening out of five potential HER2-positive breast cancer that were confirmed to have *ERBB2* gene amplification by FISH and HER2 protein expression by Western blotting (Fig. S1), on the basis of sensitivity to T-DM1 and insensitivity to the cell-autonomous effects of trastuzumab, broadly matching the clinical setting in which T-DM1 is used (Fig. S2). All cell lines displayed hallmarks of DNA aneuploidy, consistent with extensive copy number variation described in HER2-enriched carcinoma [55] without any evidence of subclonal ploidy variations (Fig. S3).

The GeCKOv2 lentiviral library of 123,411 sgRNA targeting 19,050 genes and 1,000 non-targeting control sgRNA was transduced into MDA-MB-453 and MDA-MB-361 Cas9-expressing cell lines at a measured multiplicity of infection of 0.28 and 0.29, respectively. Cas9-expressing MDA-MB-453 and MDA-MB-361 were transduced with the GeCKOv2 library at scale and subjected to protracted selection with T-DM1 or DM1. The drugs were administered at escalating concentrations in an on–off schedule (Fig. 1B) to establish growth differentials of at least 10^2^ relative to controls but without substantial cell kill to prevent stochastic sgRNA extinction and thus maintain high sgRNA representation (Fig. 1C).

At the conclusion of the screens, cell proliferation assays were conducted to assess the emergence of bulk drug resistance. MDA-MB-453 libraries challenged with T-DM1 had a 3.7-fold greater T-DM1 IC_50_ than control-treated libraries, while there was no change in DM1 IC_50_ between DM1- and control-treated cells (Fig. 1D). Modest bulk resistance to T-DM1 with a 2.4-fold increase in IC_50_ was observed in MDA-MB-361 libraries exposed to this agent relative to untreated libraries, while a 1.8-fold increase in DM1 IC_50_ was observed in MDA-MB-361 libraries exposed to DM1 relative to controls (Fig. 1E). Neither the T-DM1 nor DM1-treated MDA-MB-453 or MDA-MB-361 libraries developed cross-resistance to the HER2-targeted small molecule kinase inhibitors lapatinib and neratinib (Fig. S4). Notably, in T-DM1 and DM1-treated MDA-MB-361 cultures, further treatment with high concentrations of T-DM1 or DM1, respectively, did not inhibit viability of all cells (Fig. 1E) indicating the presence of resistant clones. To investigate further, T-DM1-treated and T-DM1-naïve MDA-MB-361 libraries were exposed to T-DM1 and assessed for clonogenic survival after 21 days. A greater number of surviving clonogens was observed in the libraries challenged with T-DM1 relative to T-DM1-naïve libraries (Fig. S5).

To evaluate if resistance to T-DM1 may have arisen through loss of expression or truncation of HER2, which are reported resistance mechanisms to HER2-targeted therapies [22, 26, 34, 39], immunoblotting for HER2 was carried out at the conclusion of the screens in MDA-MB-453 and MDA-MB-361 GeCKOv2 lysates using a monoclonal antibody that binds to the intracellular domain of HER2. T-DM1- or DM1-treated cultures showed no apparent differences in HER2 expression or molecular weight (indicated by electrophoretic mobility) when compared to untreated cultures (Fig. 1F), suggesting that CRISPR-independent changes in HER2 epitope presentation or expression were unlikely to be major determinants of the bulk resistance that emerged during the drug screens.

### Deconvolution of whole-genome CRISPR screens

Genomic DNA from treated and untreated replicate cultures from all four screens was amplified and sequenced for analysis of sgRNA enrichment and depletion using the MAGeCK algorithm. Analysis of the sgRNA coverage for each gene target revealed a highly complex control library, where ~ 70% of genes had representation of the complete set of six sgRNAs and < 7% had fewer than four sgRNA detectable (Fig. S6A). Saturation of sequencing analysis revealed that sgRNA representation was high across samples with ≥ 108,000 sgRNA detected at a depth of 10 million reads in all samples, except one T-DM1 replicate (94,000 sgRNA) in the MDA-MB-453 screen (Fig. S6B). Principal component analysis demonstrated that the three MDA-MB-453 libraries for each treatment condition were closely related in terms of sgRNA representation, whereas the drug-treated MDA-MB-361 libraries displayed greater variation (Fig. S6C). Distribution of sgRNA read counts was similar across samples in all screens with some sgRNA enriched or depleted in DM1 and T-DM1-treated libraries relative to controls (Fig. S6D). Both libraries showed depletion of the majority of known essential genes (Fig. S6E).

**Fig. 2:**
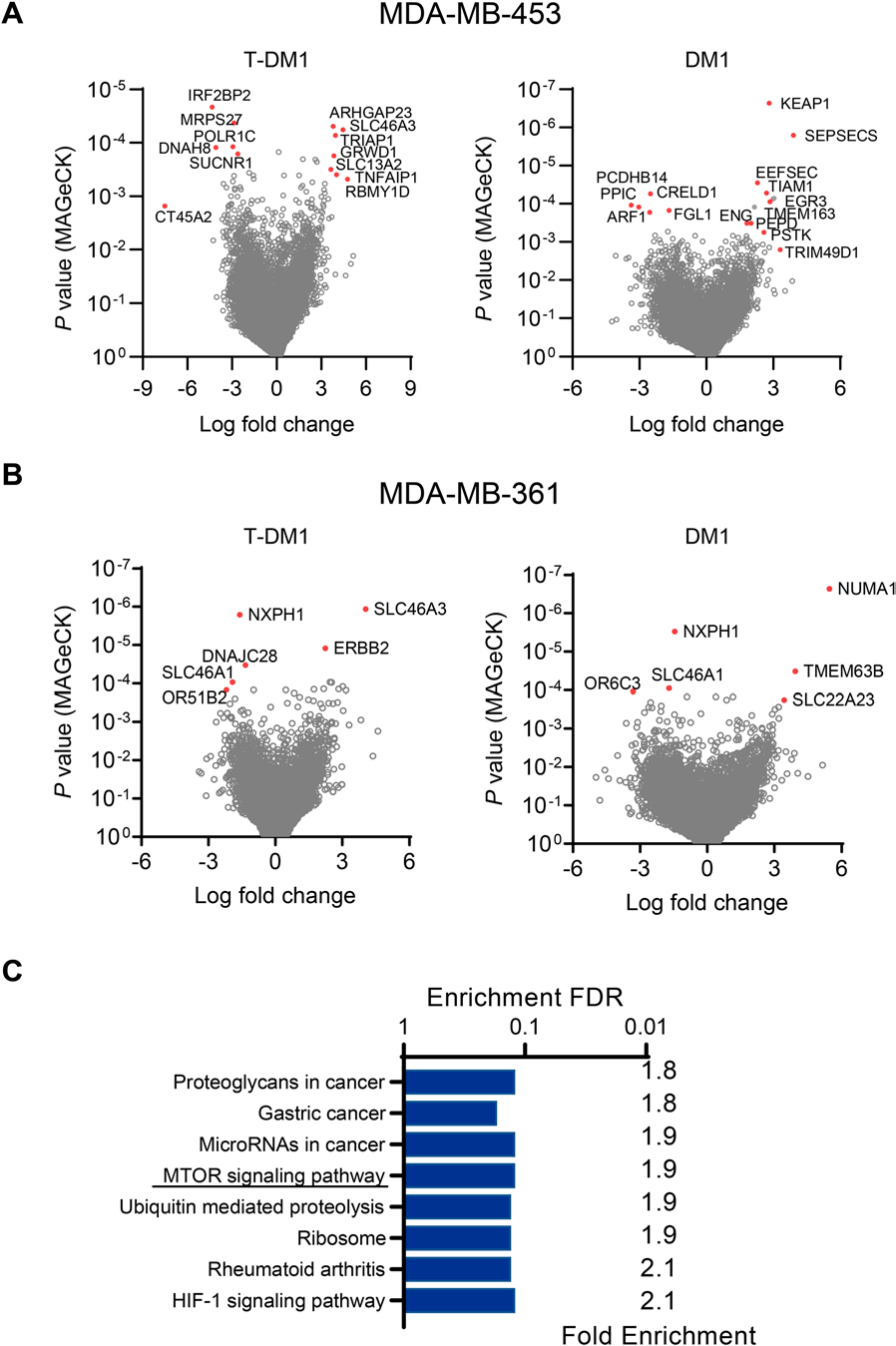
Identification of enriched or depleted genes in T-DM1 and DM1 whole-genome CRISPR/Cas9 knockout screens. **A** Volcano plots in MDA-MB-453 and **B** MDA-MB-361 cells indicating genes that were positively (sgRNA knockouts depleted) or negatively (sgRNA knockouts enriched) selected following T-DM1 or DM1 treatment in the knockout screens based on median log_2_-fold change in the representation of sgRNA against each gene in drug-treated cultures relative to untreated cultures. The statistical significance of each gene was determined using the MAGeCK statistical algorithm. Select high-ranking findings are highlighted. **C** Gene ontology (GO) analysis of the gene pathways selected negatively (knockouts thereof enriched) at *P* ≤ 0.05 (MaGeCK) in the course of both screens with T-DM1. No gene pathways were positively selected across both screens. False discovery rate (FDR) is based on nominal *P*-value from the hypergeometric test. Numerical values corresponding to a pathway report fold enrichment that is the number of genes that were selected divided by all genes in the pathway

Deconvolution of screens using the MAGeCK algorithm revealed the individual genes that potentially impacted drug response. A large number of gene knockouts (407 on average per screen at *P* < 0.01) were significantly enriched or depleted following treatment with T-DM1 or DM1 in MDA-MB-453 or MDA-MB-361 libraries (Fig. 2A,B). Gene ontology analysis performed on pooled data for MDA-MB-361(Fig. S7A) and MDA-MB-453 (Fig. S7B) identified mTOR pathway, protein ubiquitination and ribosomal genes to be overrepresented among hits (i.e., knockouts of the genes involved were enriched in T-DM1-treated cultures; Fig. 2C). Analysis of functionally interacting protein networks among treatment-enriched (Fig. S7C) or depleted (Fig. S7D) gene knockouts revealed nodes relating to microtubule and actin cytoskeleton dynamics, vesicle trafficking, RHO, JAK-STAT and mTOR signalling, cell cycle progression and apoptosis.

### High-throughput validation of hits in secondary screens

**Fig. 3:**
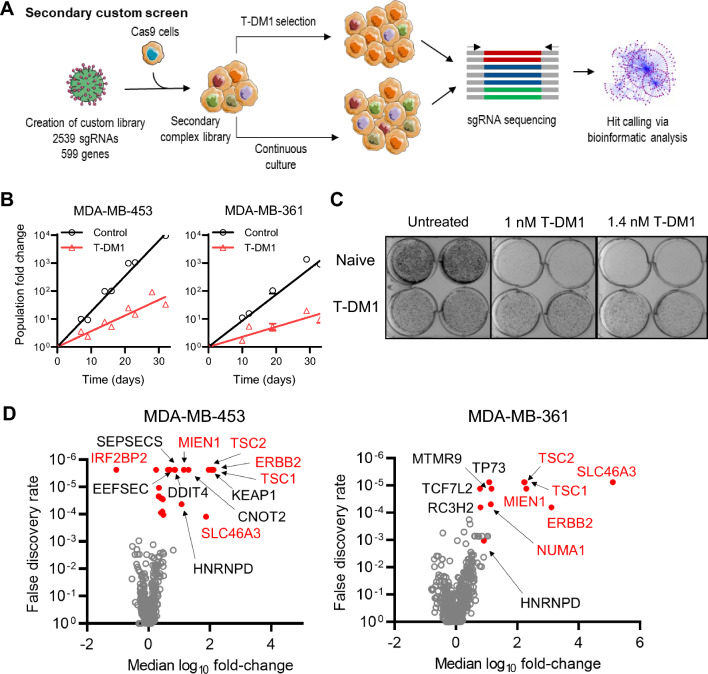
Secondary CRISPR/Cas9 knockout screens using a focused library to target 599 candidate T-DM1 sensitivity/resistance genes. **A** Schematic of the secondary screens. **B** Cell growth for the control and T-DM1-treated cultures during the screens. Each condition was performed with duplicate cultures derived from a single (MDA-MB-361) or distinct (MDA-MB-453) founder libraries and the mean growth curves are plotted. **C** Emergence of resistance to T-DM1 at the end of the screens (day 32) in cultures that were treated with T-DM1 during the screens relative to untreated (naïve) cultures in MDA-MB-361 cells, as assessed by clonogenic survival of cultures exposed continuously to 1 or 1.4 nM T-DM1 over 21 days. **D** Volcano plot of the statistical significance of gene-level enrichment or depletion as a function of the median log_2_ fold-change in normalised read counts (T-DM1/control) of all sgRNA against each target (4 sgRNA per gene) in all replicate screens performed in MDA-MB-361 and MDA-MB-453 cells

From the analyses of the whole-genome screens, as well as genes potentially implicated in T-DM1 resistance based on literature, 599 genes were selected as potential modifiers of T-DM1 or DM1 sensitivity for high-throughput validation in secondary CRISPR/Cas9 screens (Fig. 3A). A focused library of 2539 sgRNA was generated to target the 599 genes (Table S1) with four sgRNA per gene plus non-targeting controls. sgRNA were sourced from the TKO3 library [56] to ensure they were independent of sequences used in the GeCKOv2 library that was utilised for the whole-genome screens. Cas9-expressing MDA-MB-453 and MDA-MB-361 cells were transduced with the focused library in single-vector format and exposed to escalating concentrations of T-DM1 for approximately 30 days to induce growth differentials of > 100-fold (Fig. 3B). At the conclusion of the MDA-MB-361 screens, cells were more resistant to T-DM1 than untreated libraries (Fig. 3C; MDA-MB-453 cultures not tested). Library representation was high across all screen samples (Fig. S8A,B) with similar representation and read count distribution across related samples (Fig. S8C,D), while depletion of included essential genes was observed in all libraries (Fig. S8E). Voom/limma/ROAST analysis of sgRNA sequencing revealed 15 genes that were significantly enriched or depleted in the MDA-MB-453 screen and 10 genes in the MDA-MB-361 screen at *P* < 0.001 (Fig. 3D), all but five of which were also significantly enriched or depleted in the T-DM1 whole-genome screens (Table 1). Notably, there were five genes that were enriched with *P* < 1 × 10^–5^ in both screens (*ERBB2*, *SLC46A3*, *TSC1*, *TSC2* and *MIEN1*) and one gene that was depleted with *P* < 0.01 in both screens (*ILK*). The most highly depleted gene across both secondary screens was *IRF2BP2* (*P* = 6 × 10^–7^ in MDA-MB-453 cells), which was also the highest ranked negative-selection hit in the primary T-DM1 screen in MDA-MB-453 cells.

**Table 1:** Top ranked enriched and depleted genes in T-DM1 CRISPR/Cas9 knockout screens

	Gene	*P*-value	Rank in T-DM1 screens			
			MDA-MB-453 secondary (599 genes)	MDA-MB-453 whole genome (19,050 genes)	MDA-MB-361 secondary (599 genes)	MDA-MB-361 whole genome (19,050 genes)
Enriched genes in MDA-MB-453 secondary screen (***P*** < 0.001)						
	*ERBB2*	8.3E-08	1***	3156	2***	2***
	*TSC2*	8.3E-08	2***	167*	5***	6999
	*KEAP1*	8.3E-08	3***	952	173	4383
	*MIEN1*	4.2E-07	4***	8381	4***	121*
	*DDIT4*	5.8E-07	5***	68*	77	16,947
	*NR2F1*	2.6E-06	6***	10,392	430	20,479
	*TSC1*	3.9E-06	7***	100*	3***	1419
	*SLC46A3*	7.7E-06	8***	2***	1***	1***
	*CNOT2*	1.6E-05	9***	3134	49	85*
	*CDKN1B*	3.0E-05	10***	796*	214	13,663
	*HNRNPD*	3.8E-05	11***	10,690	16*	6**
	*TGFBR3L*	3.6E-04	12***	2917	316	5879
	*RHOA*	4.1E-04	13***	2605	221	16,163
	*EEFSEC*	9.3E-04	14***	99*	497	2680
Enriched genes in MDA-MB-361 secondary screen (***P*** < 0.001)						
	*SLC46A3*	8.3E-08	8***	2***	1***	1***
	*ERBB2*	8.3E-08	1***	3156	2***	2***
	*TSC1*	8.3E-08	7***	100*	3***	1419
	*MIEN1*	1.1E-06	4***	8381	4***	121*
	*TSC2*	2.6E-06	2***	167*	5***	6999
	*TP73*	1.5E-04	145	4910	6***	2137
	*MTMR9*	1.6E-04	380	7258	7***	158*
	*RC3H2*	2.9E-04	572	1439	8***	7**
Depleted genes in MDA-MB-453 secondary screen (***P*** < 0.001)						
	*IRF2BP2*	5.8E-07	1***	1***	360	11,738
Depleted genes in MDA-MB-361 secondary screen (***P*** < 0.001)						
	*CCAR2*	3.2E-04	19*	4831	1***	242*
	*GREB1*	7.0E-04	23*	1057	2***	6**

^*^, *P* < 0.05

^**^, *P* < 0.001

^***^, *P* < 0.0001

### Individual validation of *TSC1* and *TSC2* as T-DM1 sensitivity genes

**Fig. 4:**
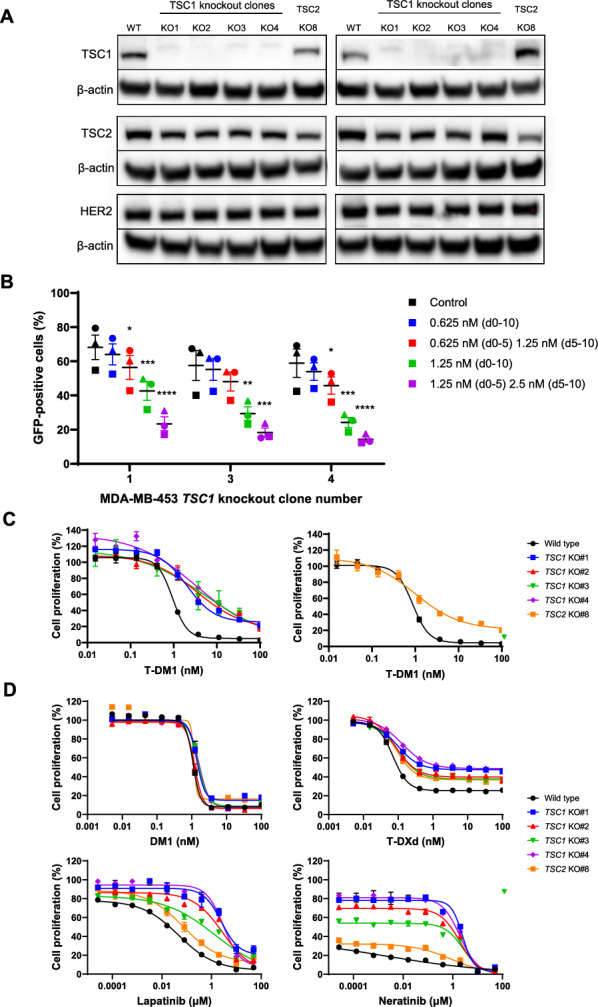
Validation of *TSC1* and *TSC2* as T-DM1 sensitivity genes. **A** Protein expression of TSC1, TSC2 and HER2 by Western blotting in *TSC1*-knockout and partial *TSC2*-knockout MDA-MB-453 clonal cell lines. Each blot represents different cell lysates. β-actin was used as a protein loading control. **B** Competition growth assay of GFP-negative *TSC1*-knockout MDA-MB-453 clonal cell lines #1, #3 and #4 cultured 1:1 with GFP-positive MDA-MB-453 wild type cells and treated with T-DM1 at the indicated concentrations for 10 days. Cells either received the same concentration of T-DM1 for 10 days (d0–10), or an initial concentration for days 0–5 that was subsequently doubled for days 5–10. Lines represent the mean ± SEM for three separate experiments, with each separate experiment represented as a different symbol. *, *P* < 0.05; **, *P* < 0.01; ***, *P* < 0.001; ****, *P* < 0.0001. **C** Growth inhibition curves of T-DM1 in *TSC1*-knockout and partial *TSC2*-knockout MDA-MB-453 clonal cell lines. **D** Growth inhibition curves of DM1, T-DXd, lapatinib and neratinib in *TSC1*-knockout and partial *TSC2*-knockout MDA-MB-453 clonal cell lines. Plots in C) and D) are representative images of growth inhibition plots of three separate experiments. Symbols represent mean ± SEM of two technical replicates

Among the top enriched hits in both secondary screens were the tuberous sclerosis complex genes *TSC1* and *TSC2*. To confirm their role as T-DM1 sensitivity genes, we attempted to generate MDA-MB-453 clonal knockout cell lines via CRISPR/Cas9 ribonucleoprotein delivery. Four MDA-MB-453 clonal cell lines were confirmed to have knockout of TSC1 by Western blotting, but only partial knockout of TSC2 was achieved in a single clonal cell line (Fig. 4A, Fig. S9). We then compared the activity of T-DM1 in knockout clones to MDA-MB-453 wild type cells in functional assays. *TSC1*-knockout MDA-MB-453 clonal cell lines were more resistant to T-DM1 than GFP-positive wild type cells in competition growth assays, indicating that *TSC1* knockout reduced sensitivity to T-DM1 (Fig. 4B). These findings were confirmed in a growth inhibition assay, where T-DM1 was less active in both *TSC1* and partial *TSC2* knockout MDA-MB-453 clonal cell lines than wild type cells, indicated by rightward shifts in the IC_50_ curves and reductions in maximal response (Fig. 4C).

To see if an altered response to DM1 or HER2 signalling might be responsible for the *TSC1* and *TSC2*-mediated sensitivity to T-DM1, we evaluated the antiproliferative activity of DM1 and the HER2-targeting therapies T-DXd, lapatinib and neratinib in MDA-MB-453 *TSC1* knockout and partial *TSC2* knockout clones. DM1 activity was largely unchanged in *TSC1* knockout and partial *TSC2* knockout clones, but T-DXd, lapatinib and neratinib were all considerably less active in the *TSC1* knockout clones and slightly less active in the partial *TSC2* knockout clone (Fig. 4D). Similar to T-DM1, there was a reduction in maximal response with high concentrations of T-DXd in the knockouts relative to the wild type cells, but not for lapatinib, neratinib or DM1. As the wild type MDA-MB-453 cells were already resistant to trastuzumab (Fig. 1E), it was not possible to evaluate further resistance to trastuzumab in the knockout lines.

**Fig. 5:**
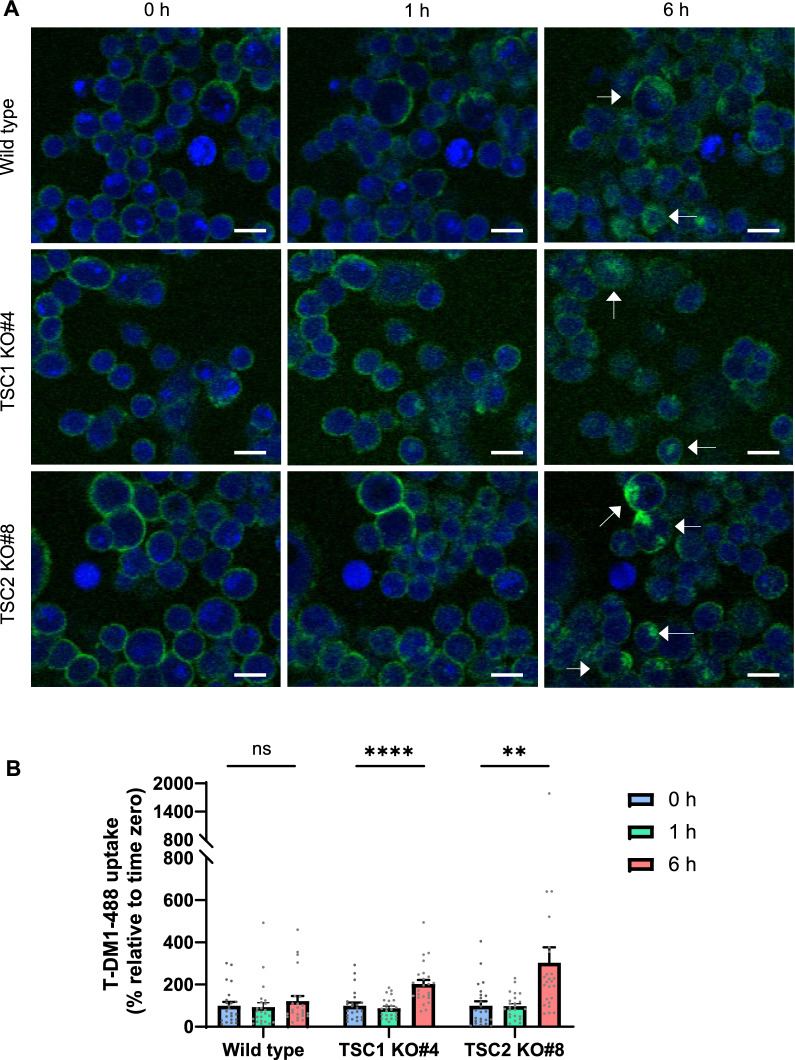
Internalisation of T-DM1 increases with *TSC1* knockout and partial *TSC2* knockout. MDA-MB-453 wild type, *TSC1* knockout (clone #4) and *TSC2* partial knockout (clone #8) cells were incubated with T-DM1 conjugated to Alexa Fluor 488 (T-DM1-488) and imaged as live cells over time by confocal microscopy. **A** Confocal microscopy images at 20 × objective. Arrows indicate cells with internalisation of T-DM1-488 into the cytoplasm at 6 h. Scale bar = 20 µm. **B** T-DM1-488 uptake into cells over time. Bars represent mean ± SEM of 24 tracked cells. ns, nonsignificant; **, *P* < 0.01, ****, *P* < 0.0001 at 6 h vs 0 h by one-way ANOVA

We also evaluated the impact of *TSC1* knockout and partial *TSC2* knockout on T-DM1 internalisation. Cells were treated with T-DM1 conjugated to Alexa Fluor 488 (T-DM1-488) and imaged by confocal microscopy. Individual cells were tracked for their uptake of T-DM1-488 over a 6 h period. Immediately after treatment, T-DM1-488 was localised at the plasma membrane. Minimal internalisation was observed at 1 h, but by 6 h, T-DM1-488 was present within the cells with colocalisation with lysosomes evident (Fig. 5A, Fig. S10). Increased T-DM1-488 internalisation was observed at 6 h in *TSC1* knockout (202% increase from 0 h, *P* < 0.0001, one-way ANOVA) and *TSC2* partial knockout (304% increase from 0 h, *P* < 0.01) cells compared to wild type cells (31% increase from 0 h) (Fig. 5B).

**Fig. 6:**
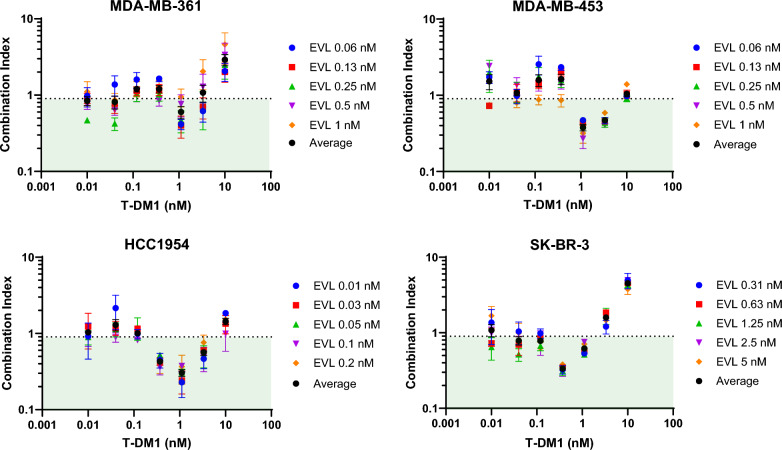
Everolimus has synergistic antiproliferative activity with T-DM1 Combination indices of antiproliferative activity of T-DM1 and everolimus. Everolimus (EVL) was tested at different concentrations to cause 20–80% inhibition of cell growth. Average indicates the average combination index across all five everolimus concentrations. Symbols represent the mean ± SEM of n = 2–4. The green box indicates synergy with combination index < 0.9

As *TSC1* and *TSC2* are negative regulators of mTOR, their role can be phenocopied by mTOR inhibition. Therefore, to evaluate if pharmacological inhibition of mTOR could increase sensitivity to T-DM1, we evaluated the growth inhibitory activity of T-DM1 in combination with the mTORC1 inhibitor everolimus in four HER2-positive breast cancer cell lines (Fig. S11). Synergy was observed across multiple everolimus concentrations in all four cell lines tested as evidenced by combination index values ≤ 0.9, but generally only at T-DM1 concentrations near and immediately above its single agent IC_50_ (Fig. 6).

## Discussion

T-DM1 is an effective therapy for HER2-positive breast cancer, but its clinical activity is limited by acquired and intrinsic resistance. Using whole-genome and focused sgRNA libraries, we conducted CRISPR screens that identified several candidate genes of T-DM1 sensitivity and resistance, including known T-DM1 sensitivity genes *ERBB2* and *SLC46A3* and new candidates *TSC1* and *TSC2*. Individual targeting of *TSC1* and *TSC2* using sgRNA validated our screening approach with the knockouts being more resistant to T-DM1 treatment, while synergistic activity for T-DM1 and the mTOR inhibitor everolimus suggests our approach may uncover potential combination strategies for overcoming T-DM1 resistance.

Among the myriad of proposed mechanisms of T-DM1 resistance [21], changes in HER2 function through reduced expression [22, 26, 34, 39], heterogeneity [24, 25] or altered binding [26], are some of the most well studied. After all, the mechanism of T-DM1 uptake requires binding to HER2 before it can be internalised and activated intracellularly [57]. Accordingly, target expression is a major determinant of sensitivity to T-DM1 and, therefore, *ERBB2* knockouts would be expected to have considerably reduced T-DM1 activity, which is exactly what we observed in both our secondary screens. While we are not aware of CRISPR/Cas9 knockout screens having previously been carried out with T-DM1, a similar screen using an anti-CD22 based ADC incorporating a DM1-like maytansine payload also had the target antigen (*CD22*) as a highly enriched hit [58], along with the lysosomal transporter *SLC46A3*, which also featured prominently in a T-DM1 shRNA screen [35]. That *ERBB2* and *SLC46A3* were among the strongest hits in our screens gave us confidence in the validity of our screening approach.

It was notable that some of our most prominent hits in the secondary T-DM1 screens were not significant in both the primary T-DM1 screens. *ERBB2* for example ranked 1st or 2nd in the MDA-MB-453 and MDA-MB-361 secondary screens but was not a significant hit in the MDA-MB-453 primary screen. Our whole-genome screens utilised the GeCKOv2 whole-genome library [59], which has subsequently been found to have inferior performance to newer generation whole-genome sgRNA libraries, with many nonfunctional or multi-targeting sgRNA [60]. Despite most sgRNA being detected in cultures in the primary screens and most essential genes being depleted (Fig. S7), only 3 of 6 sgRNA targeting *ERBB2* in the MDA-MB-361 library and 2 of 6 in the MDA-MB-453 library were enriched following T-DM1 treatment, with one sgRNA showing significant depletion in both libraries. In comparison, all four *ERBB2*-targeting sgRNAs were significantly enriched in the secondary screens, suggesting that the reduced performance of the GeCKOv2 library may have impacted our ability to identify every T-DM1 sensitivity and resistance gene. Nevertheless, the primary screens enabled us to identify ~ 600 candidate T-DM1 sensitivity and resistance genes for triage to confirm in secondary screens, which used different and better performing sgRNA [56].

Two of the top novel T-DM1 sensitivity hits from the secondary screens were *TSC1* and *TSC2*. These genes have not previously been implicated in T-DM1 sensitivity; however, they negatively regulate mTOR complex 1 (mTORC1) [61], and mTOR signalling has been shown to influence trastuzumab [62, 63] and T-DM1 activity [64, 65]. Indeed, other negative regulators of mTORC1 were also highly enriched hits in our screens (*DDIT4* [66] and *RC3H2*/Roquin-2 [67]). Knockout of *TSC1* and *TSC2* knockdown promoted resistance to T-DM1, while phenocopying TSC1 and TSC2 activity through mTOR inhibition by everolimus enhanced T-DM1 activity, further suggesting that *TSC1* and *TSC2* promote sensitivity to T-DM1 and therapeutic targeting of mTOR could overcome T-DM1 resistance. This data is consistent with the recent finding that everolimus in combination with T-DM1 had superior antitumour activity to either agent alone in HER2-positive breast cancer tumour models [64]. The authors of that study conclude that the benefit of the combination is at least partially due to mTOR-dependent lysosomal processing of T-DM1 given the regulatory role of mTOR in lysosomal function [68–70] and that this combination warrants clinical evaluation [64]. They reported an increase in T-DM1 internalisation with everolimus [64], similar to the effect we observed with fluorescently-labelled T-DM1 at 6 h in our *TSC1* and *TSC2* knockout models. Any role of mTOR on lysosomal processing would also be expected to alter the activity of other ADCs, with resistance to T-DXd also observed in our *TSC1* and *TSC2* knockout models.

mTORC1 is well-established to promote cancer progression through induction of protein synthesis, cell growth, cell proliferation, angiogenesis and suppression of autophagy [71], and together with PI3K, which along with mTOR, is activated downstream of HER2 signalling is a known resistance mechanism of T-DM1 [21, 72]. Our *TSC1* knockout and *TSC2* partial knockout models were resistant to T-DM1 and T-DXd, as well as to the HER2 tyrosine kinase inhibitors neratinib and lapatinib, but not to the payload cytotoxin DM1. These results suggest that, in addition to lysosomal processing of the antibody–drug conjugate, the impact of *TSC1* and *TSC2* (and thus mTOR inhibition) on T-DM1 activity is likely to involve inhibition of HER2 downstream signalling but not altered sensitivity to the DM1 payload. Nevertheless, our data agrees with the promising activity of the T-DM1 and everolimus combination, but we do not support clinical evaluation of the combination, owing to the overlapping pneumonitis induced by both everolimus and T-DM1 [73, 74] suggesting these agents will cause increased toxicity when dosed together, as seen with trastuzumab and everolimus in combination [75, 76].

Aside from *TSC1* and *TSC2*, several other novel gene candidates of T-DM1 sensitivity and resistance were identified. *MIEN1* (*P* < 1 × 10^–6^ in both secondary screens) is a regulator of cell migration and invasion located near *ERBB2* and frequently amplified in HER2-positive breast cancer [77, 78]. It activates Annexin A2 [77], which regulates endocytosis [79]. *STXBP2* (*P* < 1 × 10^–4^ in both secondary screens) encodes the syntaxin binding protein 2 (Munc 18–2) which is involved in intracellular vesicle trafficking and exocytosis of cytotoxic granules by natural killer cells [80, 81]. Given the functions of these two genes, we speculate that they may promote internalisation [82] and intracellular trafficking [81] of T-DM1. Other prominent hits are more likely to alter the DM1-dependent activity of T-DM1. *NUMA1* (*P* < 1 × 10^–6^ in MDA-MB-361 secondary screen) encodes the nuclear mitotic apparatus protein, NuMA, that bundles microtubules together to form and maintain the mitotic spindle [83]. It was the most significant hit across the primary DM1 screens (5.46 log_2_ fold-change, *P* = 2 × 10^–7^; MDA-MB-361). *IRF2BP2* (*P* < 1 × 10^–6^ in MDA-MB-453 screen) encodes the interferon regulatory factor 2 binding partner 2, which regulates cytokine expression and represses proapoptotic transcription factors [84]. It is more frequently amplified in HER2-positive breast cancer than in other breast cancer subtypes (cBioportal.org). *SLC46A1* was a top 5 ranked hit in both the T-DM1 and DM1 primary screens in MDA-MB-361 cells (− 1.9 and − 1.7 log_2_ fold-change; *P* < 1 × 10^–4^). It encodes the proton-coupled folate transporter. It is located near *ERBB2* on chromosome 17 and is frequently co-amplified in HER2-positive breast cancer and this is associated with lower overall patient survival (cBioportal.org). It was not evaluated in the secondary screens as no specific sgRNA could be designed for the focused library. Future validation of all these genes is warranted to confirm that they influence T-DM1 sensitivity or resistance.

## Conclusions

In summary, we have conducted whole-genome and focused sgRNA library CRISPR screens in trastuzumab-resistant HER2-positive breast cancer cell lines to identify genes that promote sensitivity and resistance to T-DM1. We identified genes already known to play a role in T-DM1 resistance as well as novel genes not previously implicated in the pharmacology of this agent. These genes could provide new combination strategies to overcome resistance to T-DM1 or predictive biomarkers to identify the patients that are most likely to benefit from T-DM1 therapy. Further work is required to confirm these genes as moderators of T-DM1 activity as well as to determine if they are specific to T-DM1 or apply to other HER2-targeting therapies.

## Supplementary Information

Additional file 1.

Additional file 2.

Additional file 3.

## Data Availability

The datasets used and/or analysed during the current study are available from the corresponding author on reasonable request.
