# Supplementary material for: CRISPR screens with trastuzumab emtansine in HER2-positive breast cancer cell lines reveal new insights into drug resistance
Source: Breast Cancer Res. 2025 Mar 31;27:48. doi: 10.1186/s13058-025-02000-1 (PMC11959757; doi:10.1186/s13058-025-02000-1)
Supplement: Supplementary file 1 — Additional file 1. [file 13058_2025_2000_MOESM1_ESM.pdf]

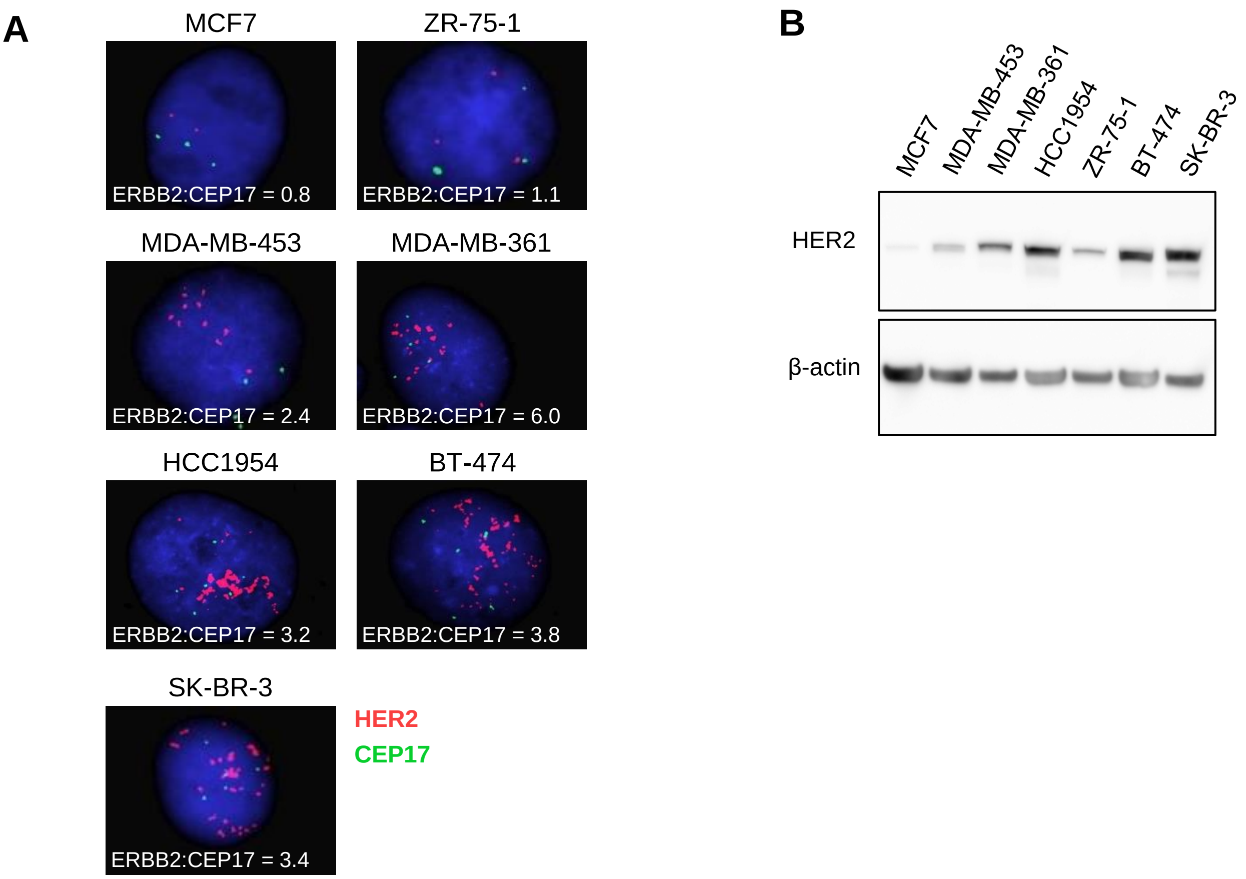

Figure S1: Assessment of HER2-positivity of breast cancer cell lines. Five HER2-positive breast cancer cell lines (MDA-MB-361, MDA-MB-453, BT-474, HCC1954, SK-BR-3) were evaluated alongside two HER2-negative or low expressing cell lines (MCF7, ZR-75-1) as controls. A) *ERBB2* gene amplification by fluorescent in situ hybridisation. Representative fluorescence micrographs (60x objective lens) of single nuclei are shown for each cell line. *ERBB2*:CEP17 ratio is indicated, estimated by the scoring of 100 cells for each cell line. An *ERBB2*:CEP17 ratio >2 indicated *ERBB2* amplification. ERBB2, red; CEP17, green; DAPI (DNA), blue. B) HER2 protein expression by Western blotting. The blot shown is representative of three separate blots of different cell lysates. β-actin was used as a protein loading control.

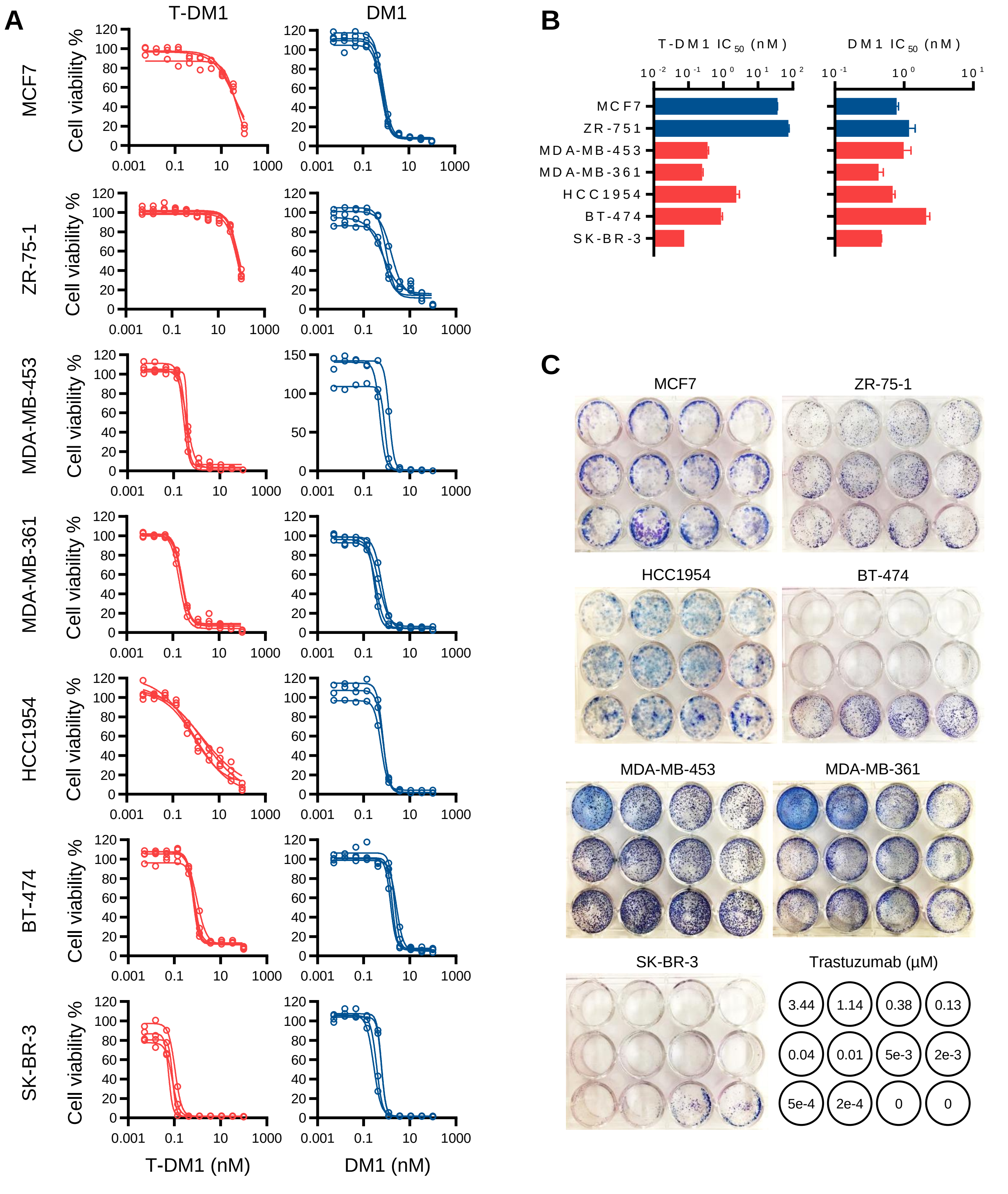

Figure S2: Sensitivity of Cas9-expressing breast cancer cell lines to T-DM1, DM1 and trastuzumab. A) Viability of cells following 6 days continuous exposure to T-DM1 (red) and DM1 (blue) by CellTiter-Glo proliferation assay. Symbols indicate biological replicates (n = 3-5; average of two technical replicates). Curves are four-parameter logistic fits for each biological replicate. B) IC_{50} values for T-DM1 and DM1 in HER2-negative or low expressing cell lines (blue) and HER2-positive cell lines (red). Bars represent mean ± SEM for n=3-5. C) Clonogenic survival after exposure to trastuzumab for 9-14 days (cell-line dependent). The photographs illustrate plates stained with methylene blue after 9-14 days (cell-line dependent) of exposure to trastuzumab. The plate layout at the lower right illustrates the drug concentrations in each well.

S3

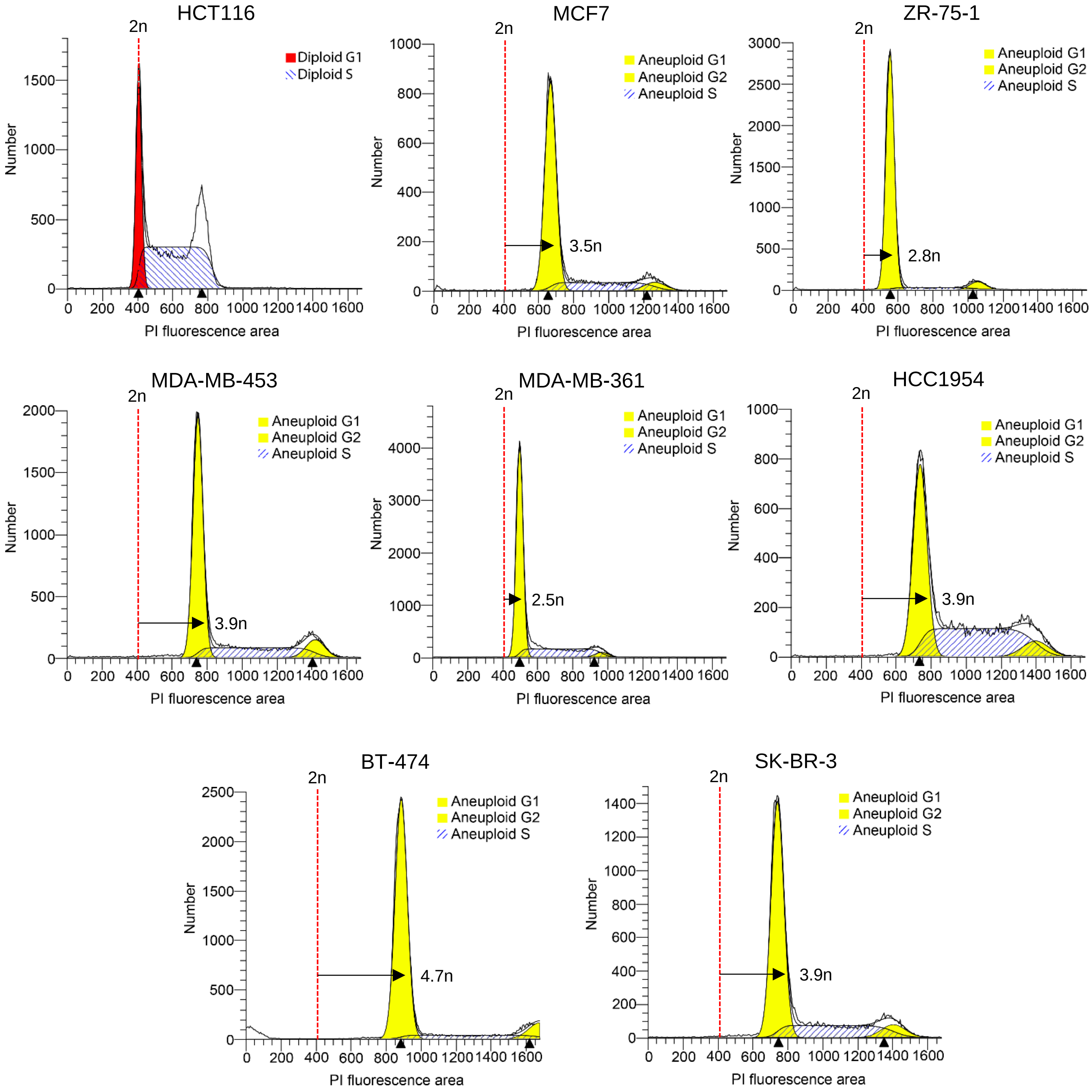

Figure S3: Ploidy analysis of the breast cancer cell line panel. Each plot depicts flow cytometry histograms illustrating the number of cells in G1, G2, or S phases of the cell cycle in propidium iodide-stained single cell suspensions derived from log-phase cultures of the panel of seven human breast cancer cell lines in addition to the near-diploid control line HCT116 as determined using ModFit. The first yellow peak in each panel represents the G1 cells and the second yellow peak represents the G2 cells. Arrowheads on x-axis indicate the modal value for G1 and G2. The dashed line represents the modal value for the G1 subpopulation in the HCT116 diploid control (2n), which was used as a reference to calculate ploidy, indicated by the arrows.

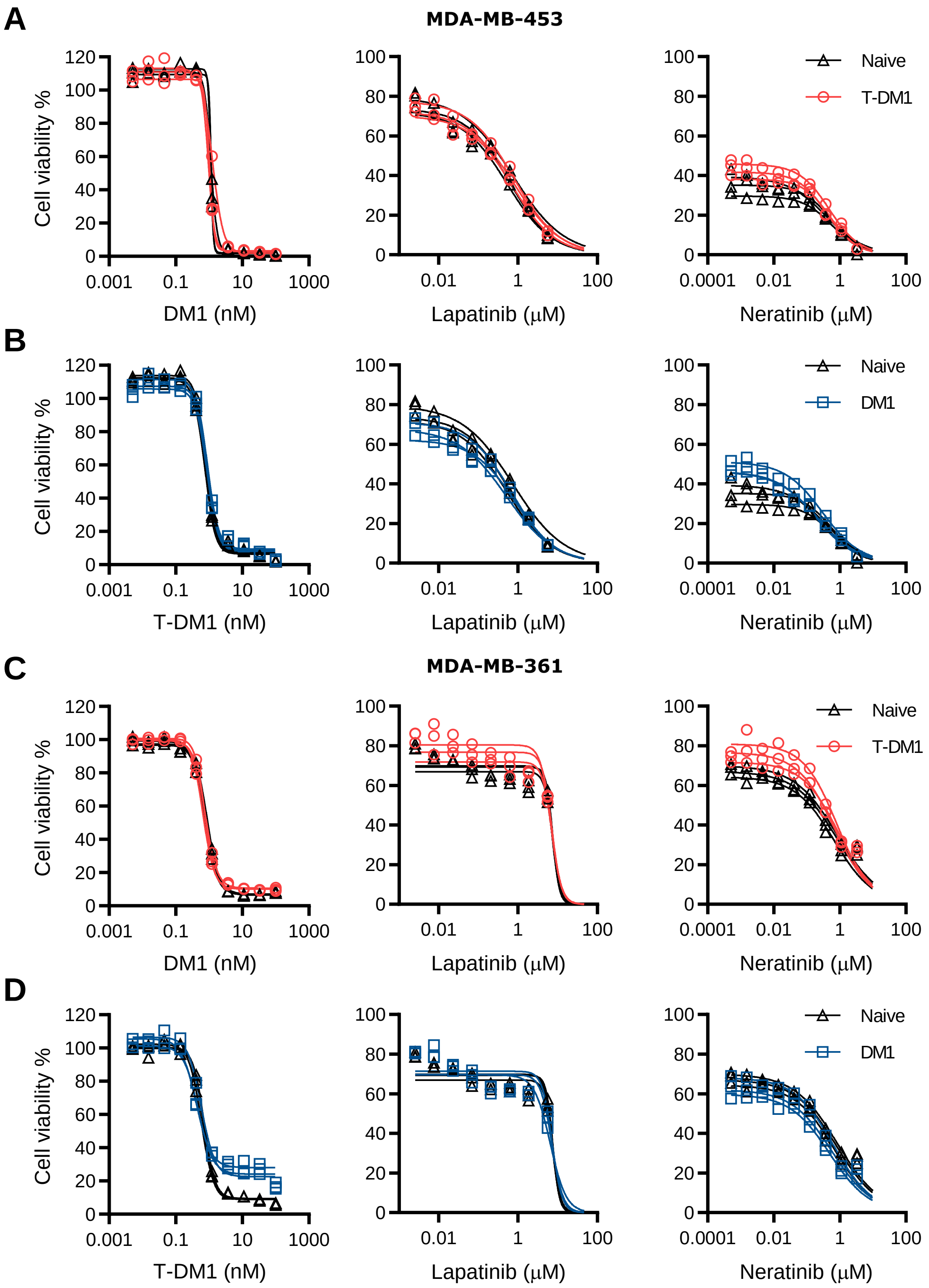

Figure S4: Lack of cross resistance of post-screen cultures to T-DM1, DM1, lapatinib and neratinib. T-DM1- or DM1-treated cultures at the conclusion of the whole genome CRISPR/Cas9 knockout screens were treated for 6 days with DM1 or T-DM1, respectively, and lapatinib and neratinib and compared to untreated (naïve) cultures from the screens treated with the same drugs. Cell viability was evaluated by CellTiter-Glo proliferation assay. A) T-DM1-treated cultures (red) and B) DM1-treated cultures (blue) from the MDA-MB-453 screens relative to untreated cultures (black). C) T-DM1-treated cultures and D) DM1-treated cultures from the MDA-MB-361 screens relative to untreated cultures. Symbols indicate biological replicates (n = 3; average of two technical replicates). Curves are four-parameter logistic fits for each biological replicate.

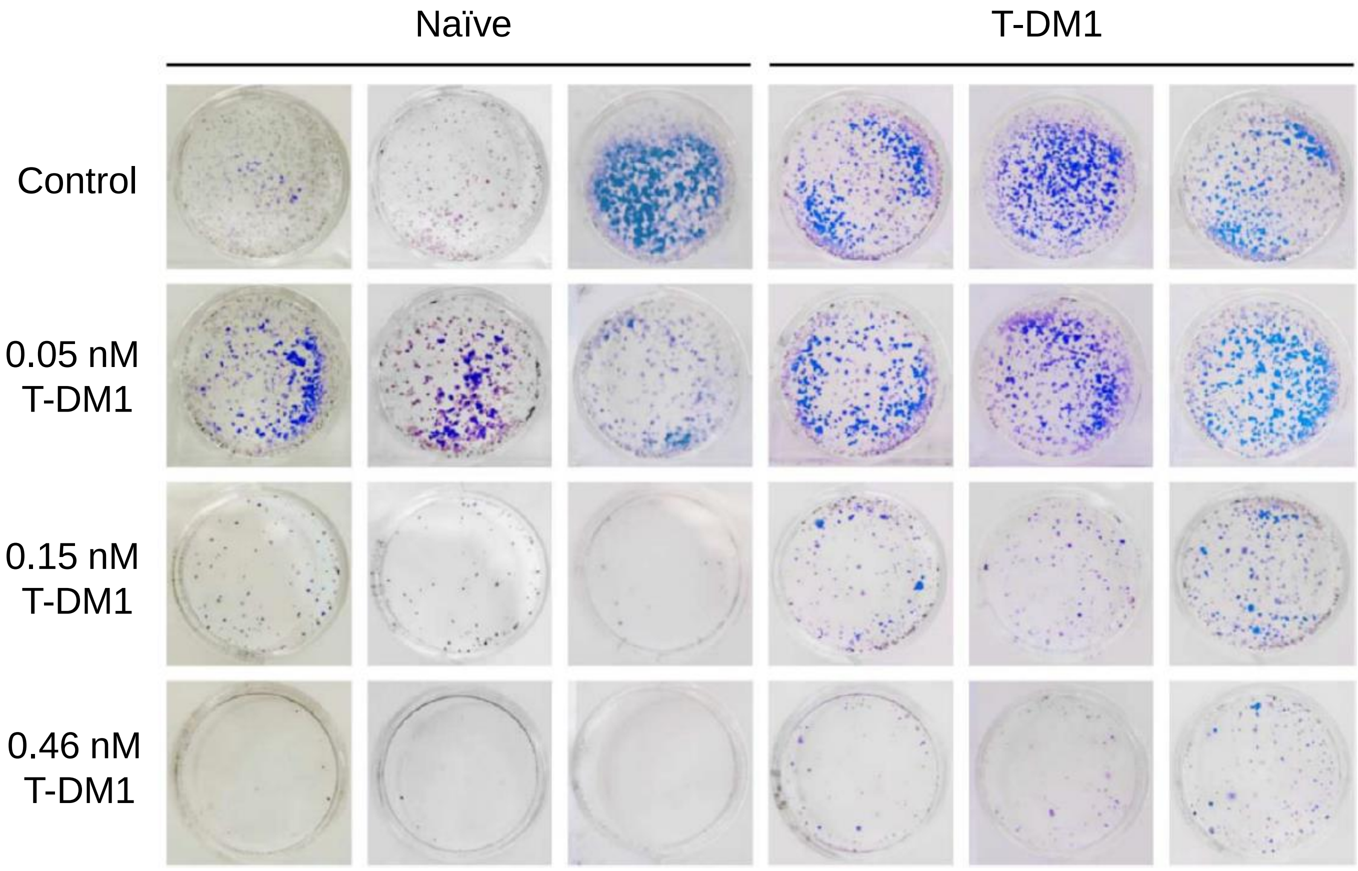

Figure S5: Sensitivity of post-screen MDA-MB-361 cultures to T-DM1. T-DM1-treated MDA-MB-361 cultures at the end of the whole genome CRISPR/Cas9 knockout screen were more resistant to T-DM1 than T-DM1-naïve cultures by clonogenic survival assay. At the end of the screen, T-DM1-treated and T-DM1-untreated cultures were exposed to T-DM1 continuously at the indicated concentrations for 21 days.

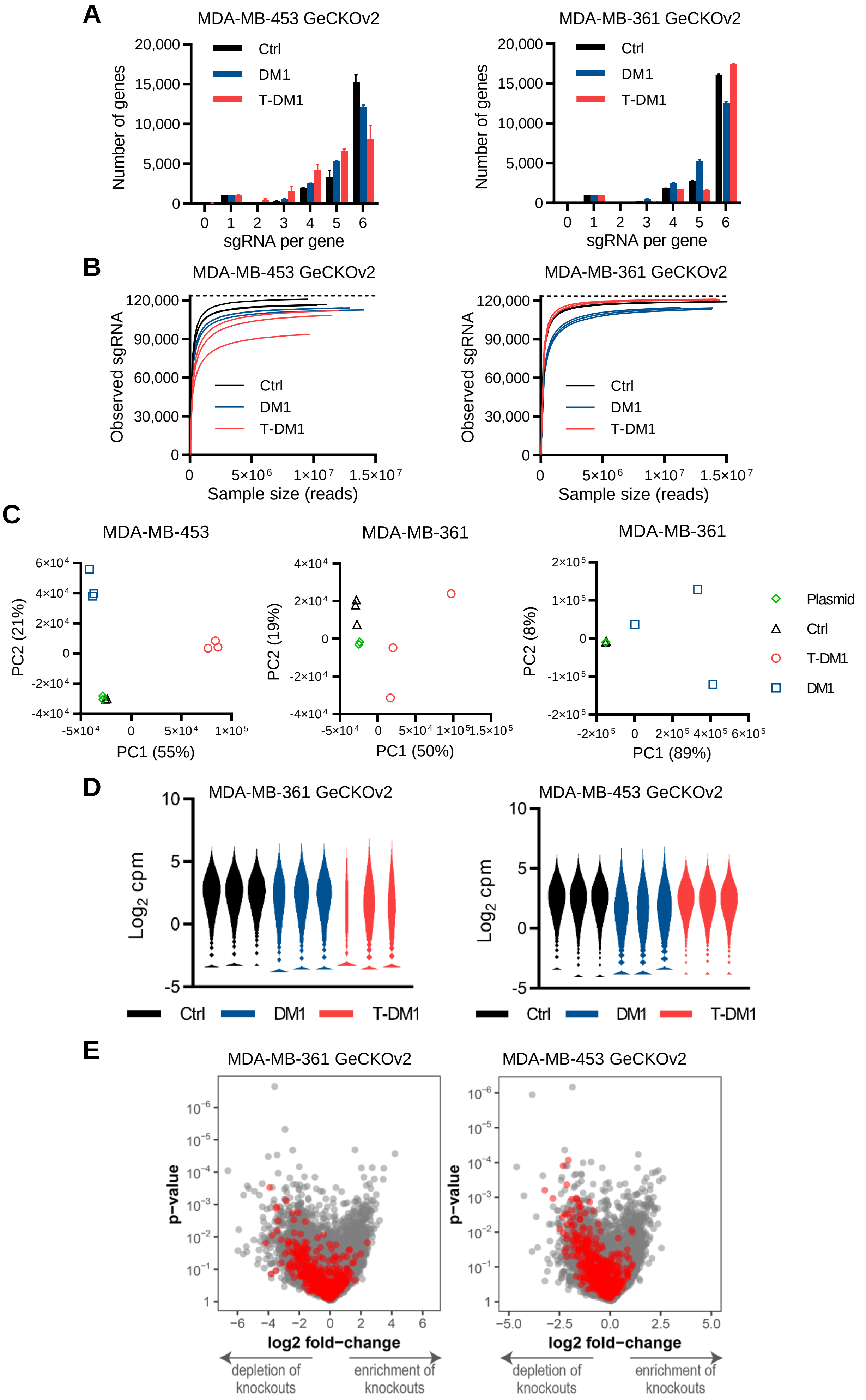
A
MDA-MB-453 GeCKOv2
MDA-MB-361 GeCKOv2
Number of genes
sgRNA per gene
Ctrl
DM1
T-DM1
B
MDA-MB-453 GeCKOv2
MDA-MB-361 GeCKOv2
Observed sgRNA
Sample size (reads)
C
MDA-MB-453
MDA-MB-361
MDA-MB-361
PC1 (55%)
PC2 (21%)
PC1 (50%)
PC2 (19%)
PC1 (89%)
PC2 (8%)
Plasmid
Ctrl
T-DM1
DM1
D
MDA-MB-361 GeCKOv2
MDA-MB-453 GeCKOv2
Log2 cpm
E
MDA-MB-361 GeCKOv2
MDA-MB-453 GeCKOv2
p−value
log2 fold−change
depletion of knockouts
enrichment of knockouts

Figure S6 (previous page): Library quality assessment for the MDA-MB-453 and MDA-MB-361 T-DM1 and DM1 whole genome CRISPR/Cas9 knockout screens. A) Coverage of sgRNA targets in the treated and control libraries. Data show the number of genes for which zero through six sgRNA were detected in the sequencing data and are the mean ± SEM of 3 biological replicates. B) Subsampling analysis of sequencing saturation. The number of unique sgRNA detected (threshold = 1 read) is plotted for random data subsamples of increasing size. The dashed line represents complete GeCKOv2 library complexity (i.e. total sgRNAs = 123,411). C) Principal component analysis of sgRNA read counts in GeCKOv2 plasmid and treated and control libraries. The scores of the first two principal components are shown for each sample, with the fraction of total variance explained by the principal component disclosed in axes titles. D) Aligned read count distributions for sgRNA in control and treated libraries. E) Ability of the MDA-MB-453 and MDA-MB-361 GeCKOv2 libraries to select for dropout of essential genes. Volcano plots are shown, with each data point showing the $\log_2$ fold-change and significance of individual sgRNAs in cells left untreated for the duration of the screens compared to the input library plasmid. Red dots indicate sgRNAs targeting essential genes, grey dots, all other genes. In both libraries, there was greater dropout (negative selection) of essential genes compared to other genes.

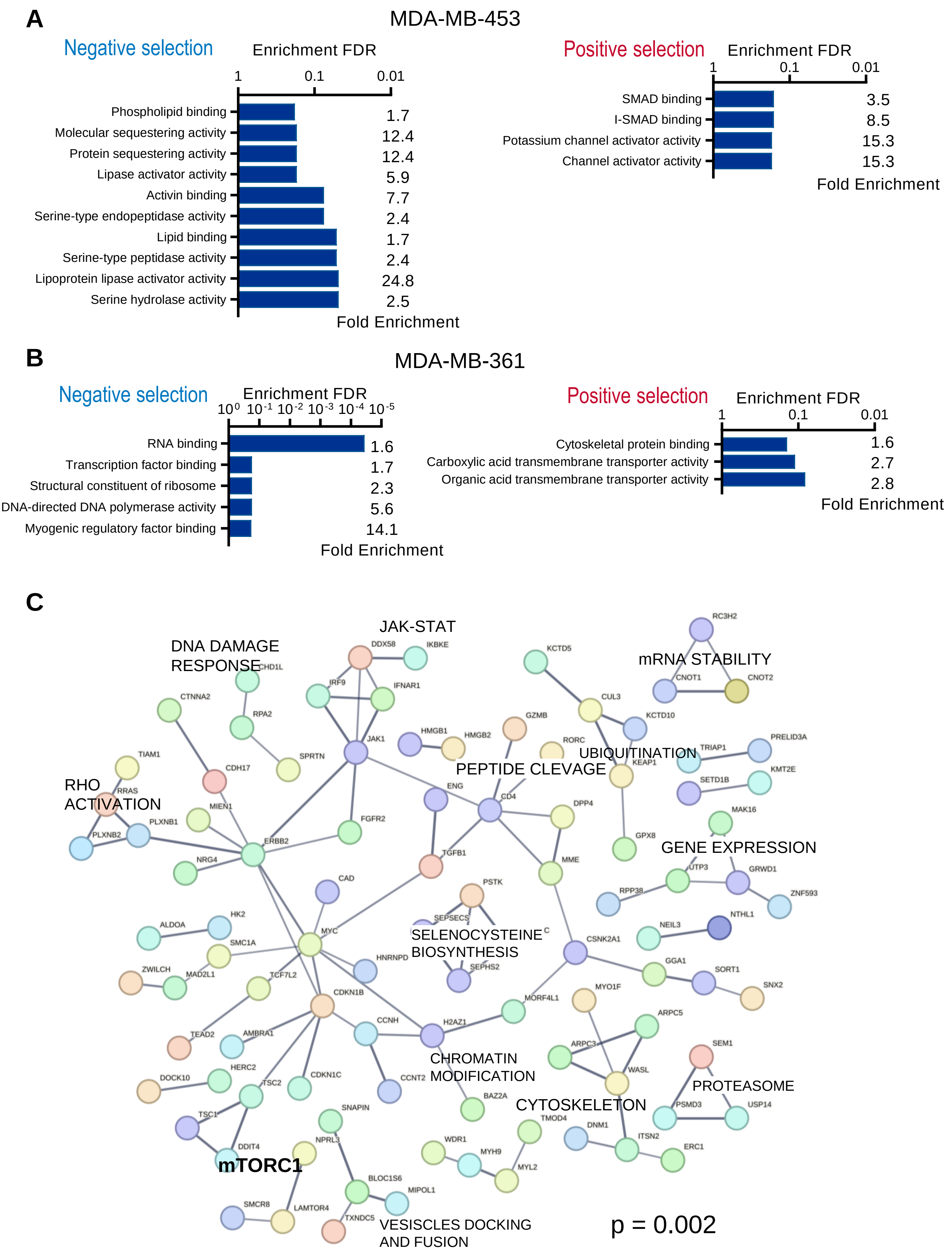
A
MDA-MB-453
Negative selection
Enrichment FDR
1
0.1
0.01
Phospholipid binding
Molecular sequestering activity
Protein sequestering activity
Lipase activator activity
Activin binding
Serine-type endopeptidase activity
Lipid binding
Serine-type peptidase activity
Lipoprotein lipase activator activity
Serine hydrolase activity
1.7
12.4
12.4
5.9
7.7
2.4
1.7
2.4
24.8
2.5
Fold Enrichment
Positive selection
Enrichment FDR
1
0.1
0.01
SMAD binding
I-SMAD binding
Potassium channel activator activity
Channel activator activity
3.5
8.5
15.3
15.3
Fold Enrichment
B
MDA-MB-361
Negative selection
Enrichment FDR
10^0 10^{-1} 10^{-2} 10^{-3} 10^{-4} 10^{-5}
RNA binding
Transcription factor binding
Structural constituent of ribosome
DNA-directed DNA polymerase activity
Myogenic regulatory factor binding
1.6
1.7
2.3
5.6
14.1
Fold Enrichment
Positive selection
Enrichment FDR
1
0.1
0.01
Cytoskeletal protein binding
Carboxylic acid transmembrane transporter activity
Organic acid transmembrane transporter activity
1.6
2.7
2.8
Fold Enrichment
C
JAK-STAT
DNA DAMAGE RESPONSE
mRNA STABILITY
RHO ACTIVATION
UBIQUITINATION
PEPTIDE CLEVAGE
GENE EXPRESSION
SELENOCYSTEINE BIOSYNTHESIS
CHROMATIN MODIFICATION
PROTEASOME
CYTOSKELETON
mTORC1
VESISCLES DOCKING AND FUSION
p = 0.002

D

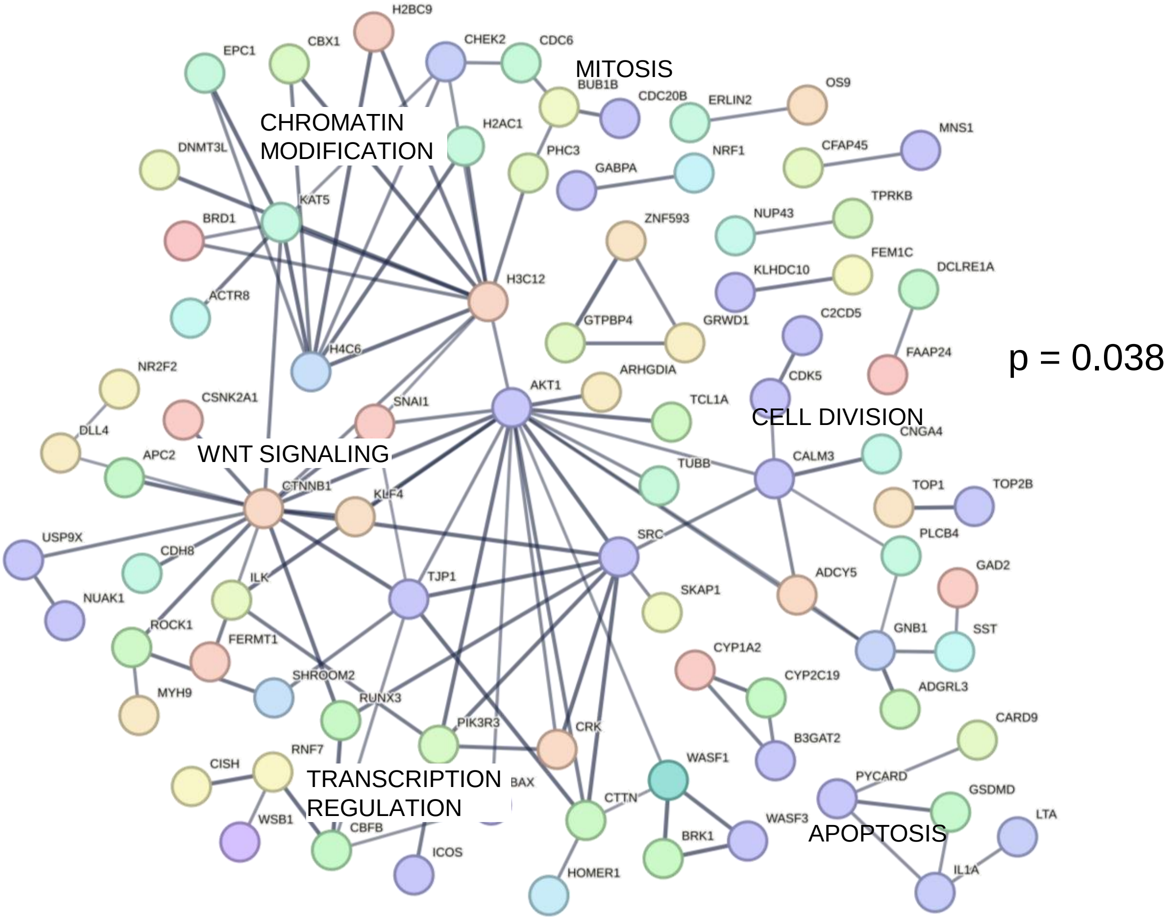

Figure S7: Gene pathways and protein networks significantly altered in response to T-DM1 treatment in MDA-MB-453 and MDA-MB-361 whole genome CRISPR/Cas9 knockout screens. A) Gene ontology (GO) analysis of the genes selected negatively (knockouts thereof enriched) or positively (knockouts thereof depleted) at P≤0.05 (MaGeCK) in the MDA-MB-453 or B) MDA-MB-361 screens with T-DM1. False discovery rate (FDR) is based on nominal P-value from the hypergeometric test. Numerical values corresponding to a pathway report fold enrichment that is the number of genes that were selected divided by all genes in the pathway. C) Analysis of functionally interacting protein networks in treatment enriched and D) depleted gene knockouts. The raw lists of screening hits were refined using the following criteria: (1) Genes with a p-value of <0.001 (tier 1) or between 0.01 and 0.001 (tier 2) identified by MAGeCK, RIGER, or PinAPL-Py deconvolution algorithms; (2) Genes selected in screens involving either DM1 or T-DM1 in MDA-MB-361 or MDA-MB-453 cells; (3) Literature evidence of gene involvement in apoptosis, the microtubule network, vesicle transport, or lysosomes, or gene amplification in >5% of breast tumours (cBioPortal). The resulting gene list was analysed using the STRING database, enabling visualisation of genes within a network context and identification of functionally enriched processes and pathways. Functional and physical associations are indicated, with line thickness representing the strength of data support. The analysis was performed at a minimum interaction score of 0.7 (high confidence) between genes. The protein-protein interaction (PPI) enrichment p-value for the analysis is shown in the frame.

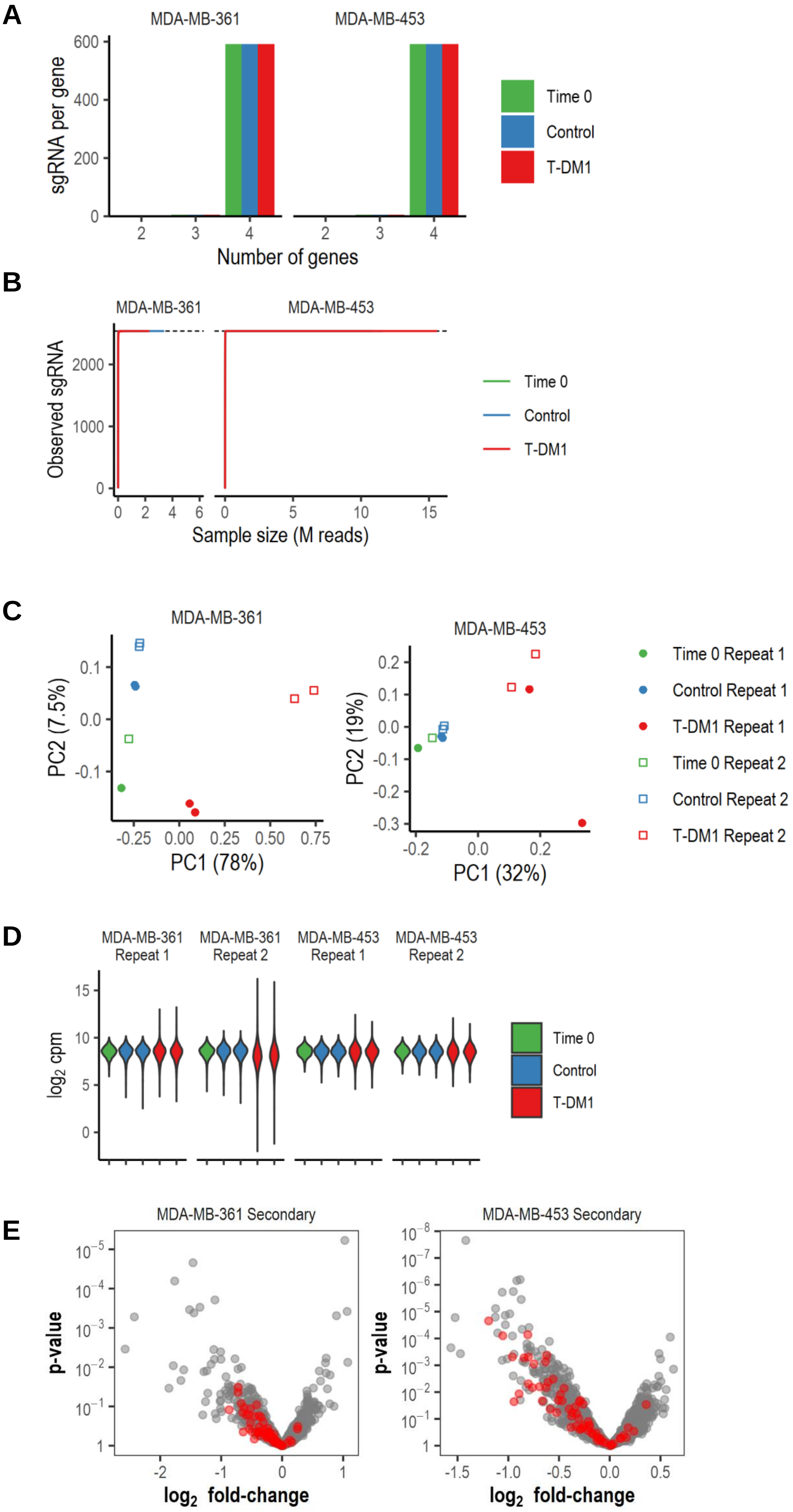

A
MDA-MB-361
MDA-MB-453
sgRNA per gene
Number of genes
Time 0
Control
T-DM1
B
MDA-MB-361
MDA-MB-453
Observed sgRNA
Sample size (M reads)
Time 0
Control
T-DM1
C
MDA-MB-361
MDA-MB-453
PC2 (7.5%)
PC1 (78%)
PC2 (19%)
PC1 (32%)
Time 0 Repeat 1
Control Repeat 1
T-DM1 Repeat 1
Time 0 Repeat 2
Control Repeat 2
T-DM1 Repeat 2
D
MDA-MB-361 Repeat 1
MDA-MB-361 Repeat 2
MDA-MB-453 Repeat 1
MDA-MB-453 Repeat 2
log2 cpm
Time 0
Control
T-DM1
E
MDA-MB-361 Secondary
MDA-MB-453 Secondary
p-value
log2 fold-change

Figure S8 (previous page): Library quality assessment for the MDA-MB-453 and MDA-MB-361 T-DM1 secondary screens. A) Coverage of sgRNA targets in the treated and control libraries. Data show the number of genes for which two through four sgRNA were detected in the sequencing data and are the mean of four biological replicates (no genes had fewer than two sgRNA detected); standard deviation is not plotted as it is below 1 for all conditions. B) Subsampling analysis of sequencing saturation. The number of unique sgRNA detected (threshold = 1 read) is plotted for random data subsamples of increasing size. The dashed line represents complete secondary library complexity (i.e. total sgRNAs = 2,539). C) Principal component analysis of sgRNA read counts in time zero, control and treated libraries. The scores of the first two principal components are shown for each sample, with the fraction of total variance explained by the principal component disclosed in axes titles. D) Read count distributions normalised to library size in control and treated libraries; zero counts given pseudocount of 0.5. E) Ability of the MDA-MB-453 and MDA-MB-361 focused libraries to select for dropout of essential genes that were included in the libraries. Volcano plots are shown, with each data point showing the log_2 fold-change and significance of individual sgRNAs in cells left untreated for the duration of the screens compared to the corresponding time zero samples. Red dots indicate sgRNAs targeting essential genes, grey dots, all other genes. In both libraries, there was greater dropout (negative selection) of essential genes compared to other genes.

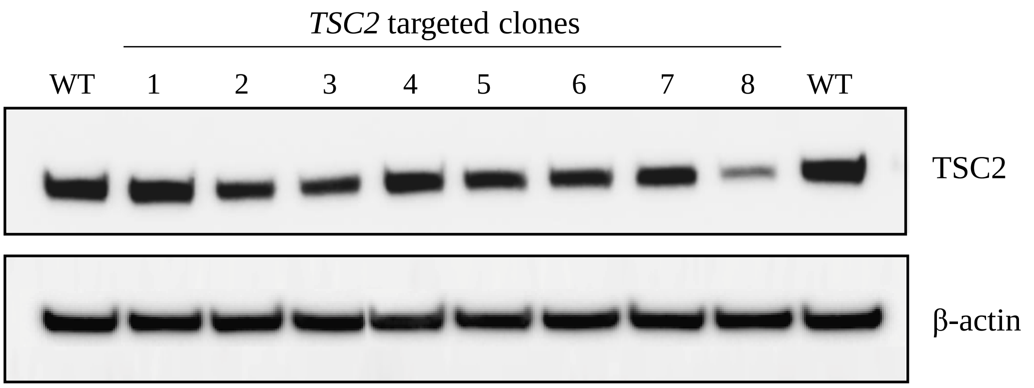

Figure S9: TSC2 protein expression in MDA-MB-453 cells targeted with sgRNA for *TSC2*. β-actin was used as a protein loading control.

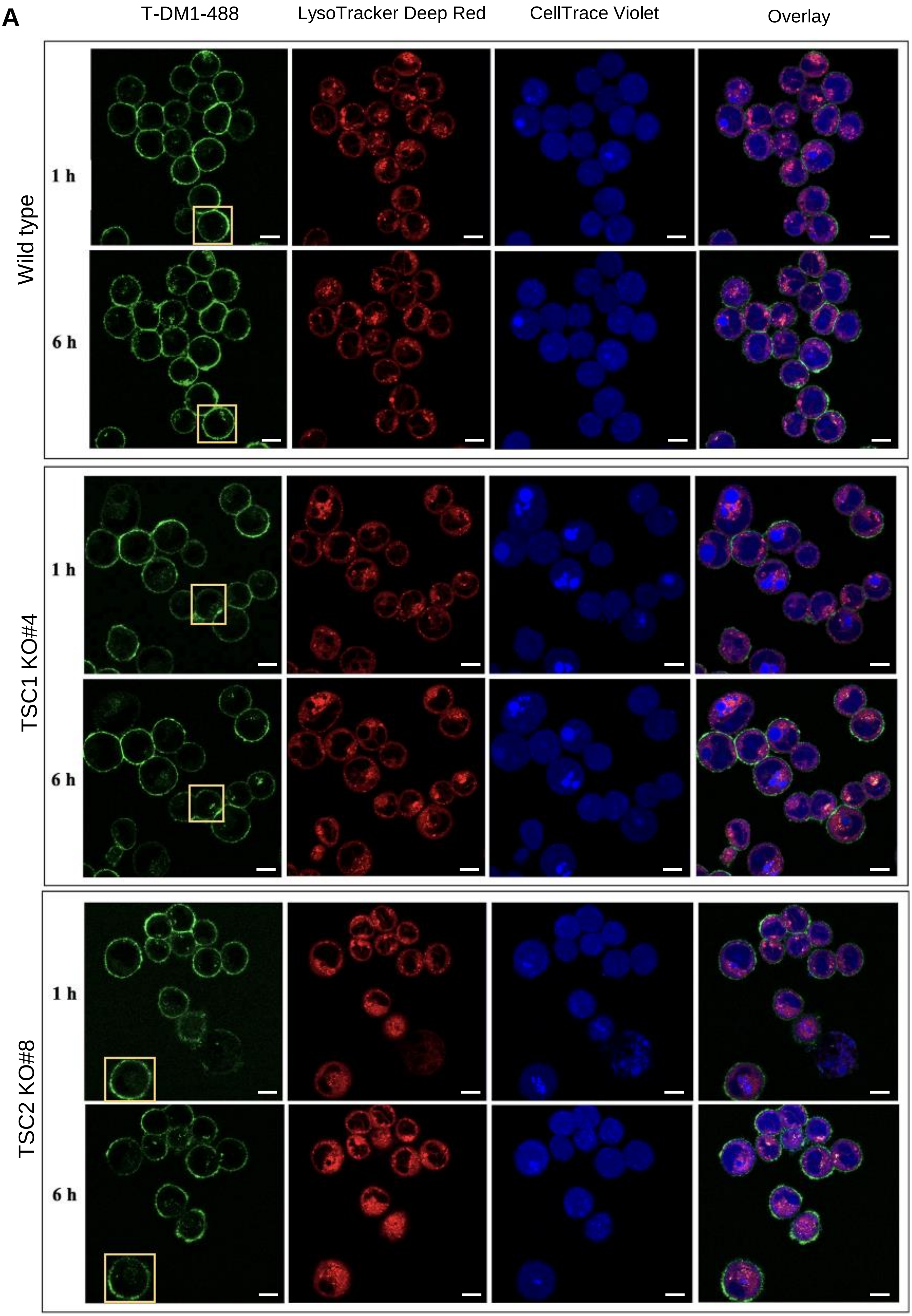
A
T-DM1-488
LysoTracker Deep Red
CellTrace Violet
Overlay
Wild type
1 h
6 h
TSC1 KO#4
1 h
6 h
TSC2 KO#8
1 h
6 h

B

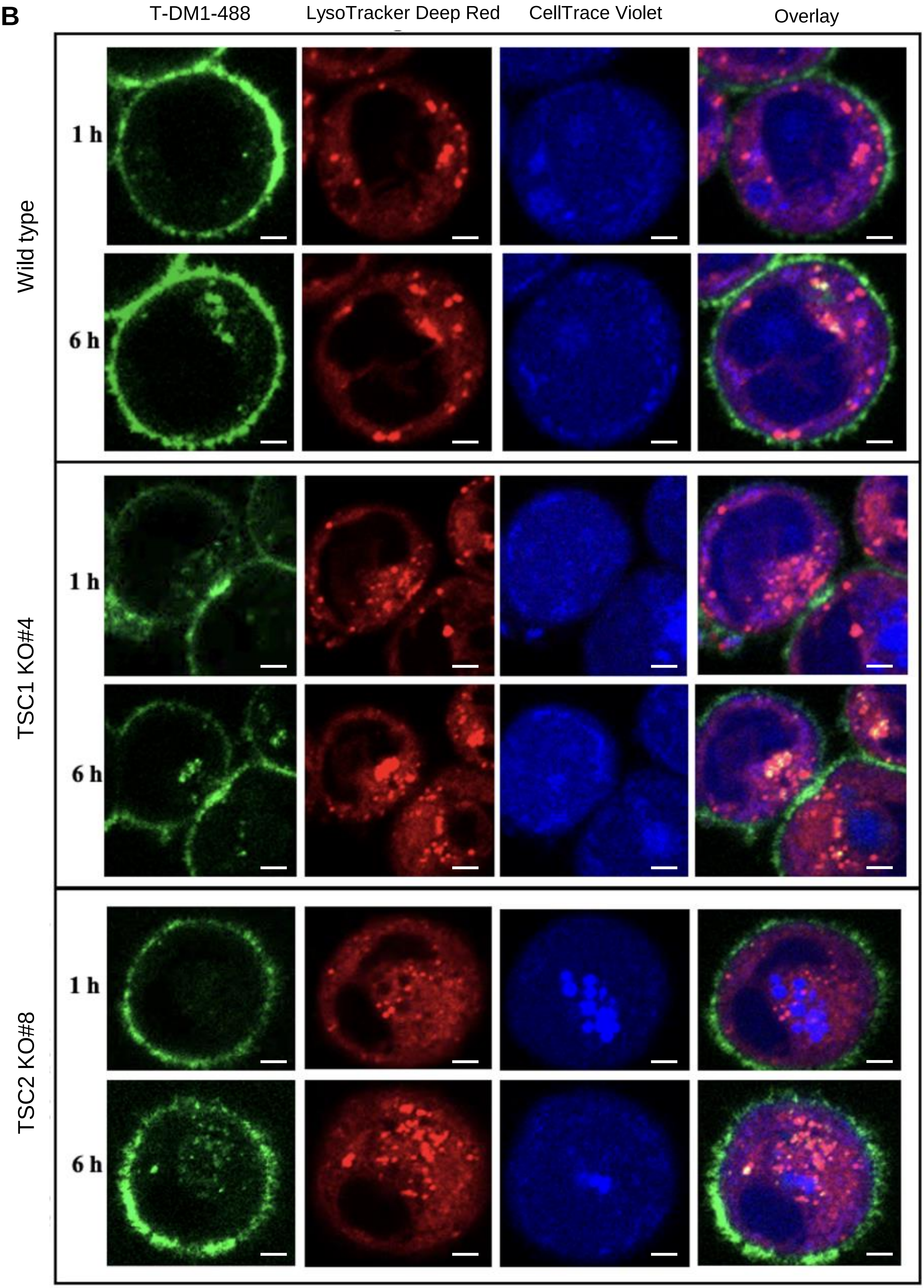
T-DM1-488
LysoTracker Deep Red
CellTrace Violet
Overlay
Wild type
1 h
6 h
TSC1 KO#4
1 h
6 h
TSC2 KO#8
1 h
6 h

Figure S10: Colocalisation of T-DM1 in lysosomes in MDA-MB-453 wild type, *TSC1* knockout and *TSC2* partial knockout cells. Cells were incubated with LysoTracker Deep Red (red) and CellTrace Violet (blue) before treatment with T-DM1 labelled with Alexa Fluor 488 (T-DM1-488, green). Images were captured at 1 h and 6 h at (A) 20× and (B) 63× magnification. Yellow boxes in (A) show the individual cells that are displayed in (B). Scale bar = 20 µm in (A) and 5 µm in (B). Yellow dots in overlay images indicate colocalisation of T-DM1-488 in lysosomes.

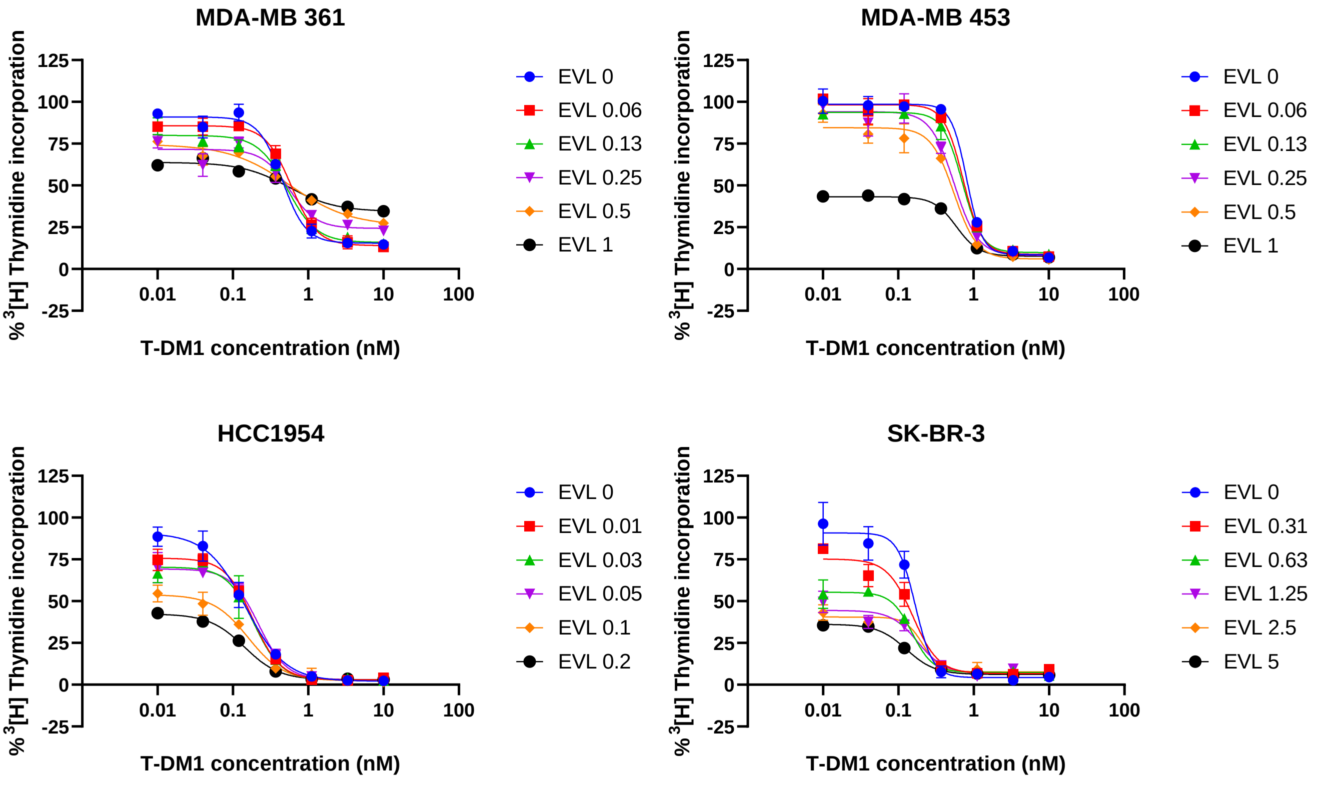

Figure S11: Antiproliferative activity of T-DM1 in combination with everolimus in four HER2-positive breast cancer cell lines. Cells were treated with continuous exposure to T-DM1 in a 3-fold dilution series and/or five different concentrations of everolimus (EVL) for 3 days and assessed for ^{3}H-thymidine incorporation. Each plot is representative of three separate experiments. Symbols represent mean ± SEM of two technical replicates.
