# Supplementary material for: CRISPR screens with trastuzumab emtansine in HER2-positive breast cancer cell lines reveal new insights into drug resistance
Source: Breast Cancer Res. 2025 Mar 31;27:48. doi: 10.1186/s13058-025-02000-1 (PMC11959757; doi:10.1186/s13058-025-02000-1)
Supplement: Supplementary file 3 — Additional file 3. [file 13058_2025_2000_MOESM3_ESM.pptx]

## Slide 1
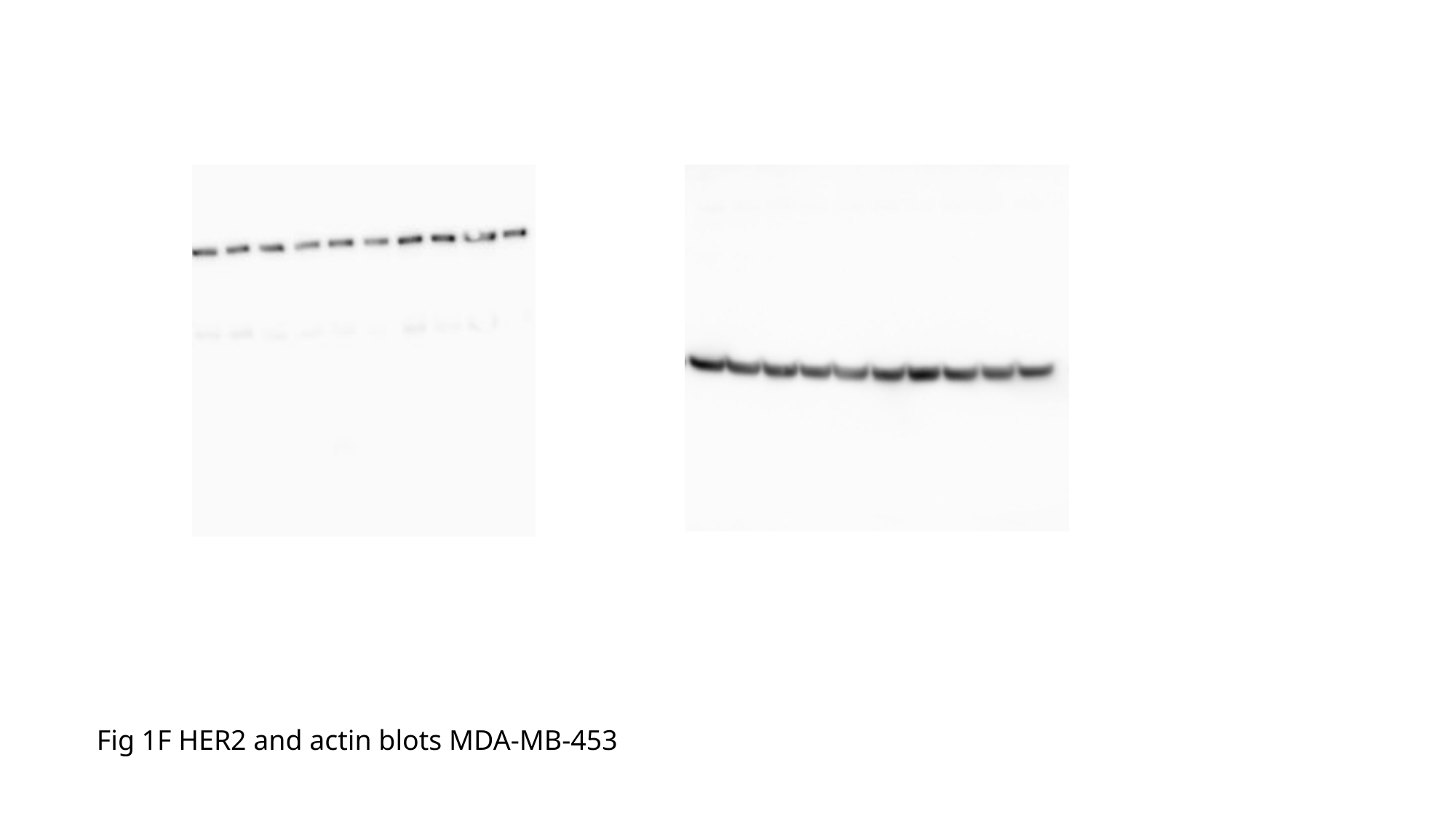

Fig 1F HER2 and actin blots MDA-MB-453

## Slide 2
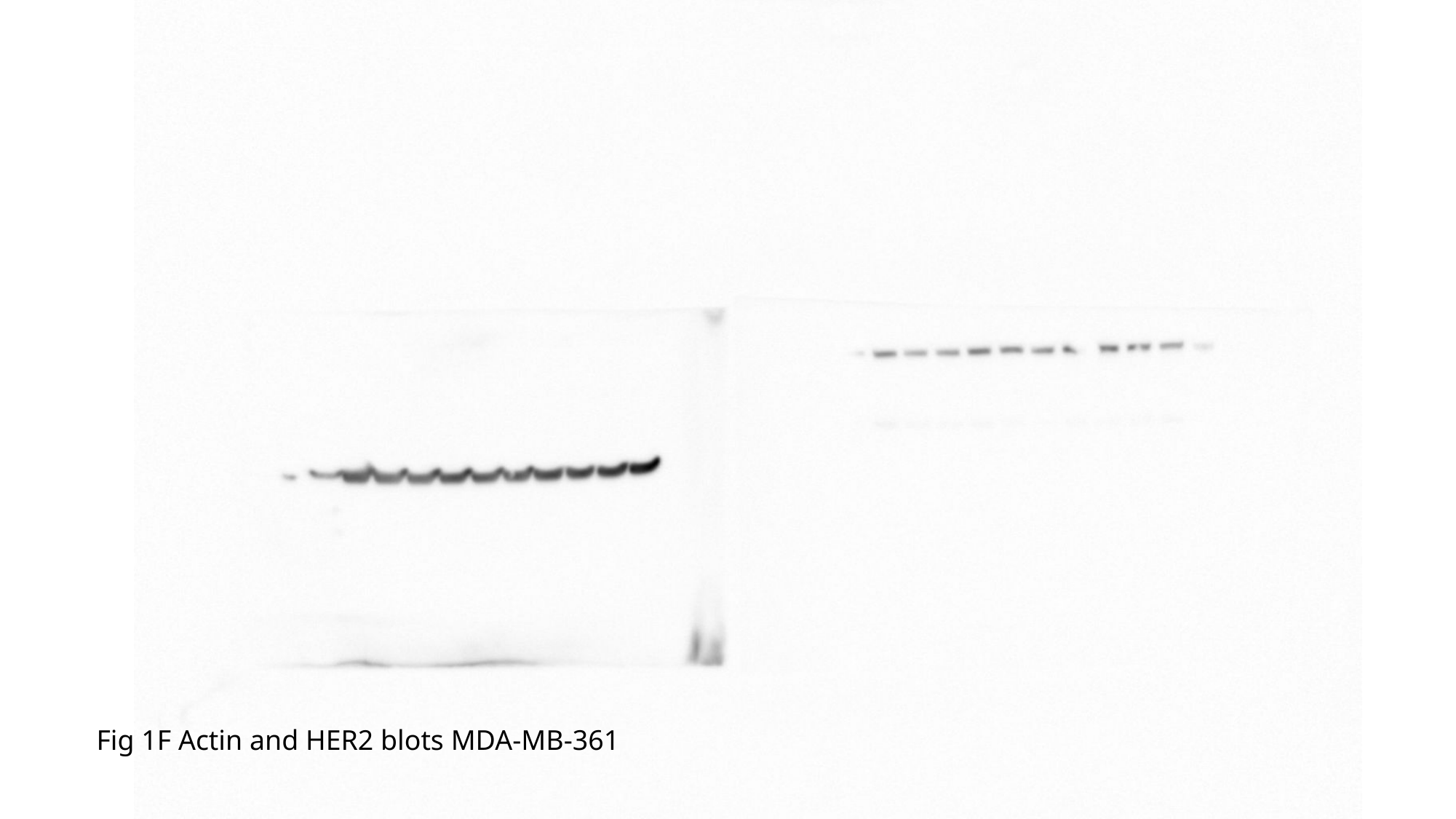

Fig 1F Actin and HER2 blots MDA-MB-361

## Slide 3
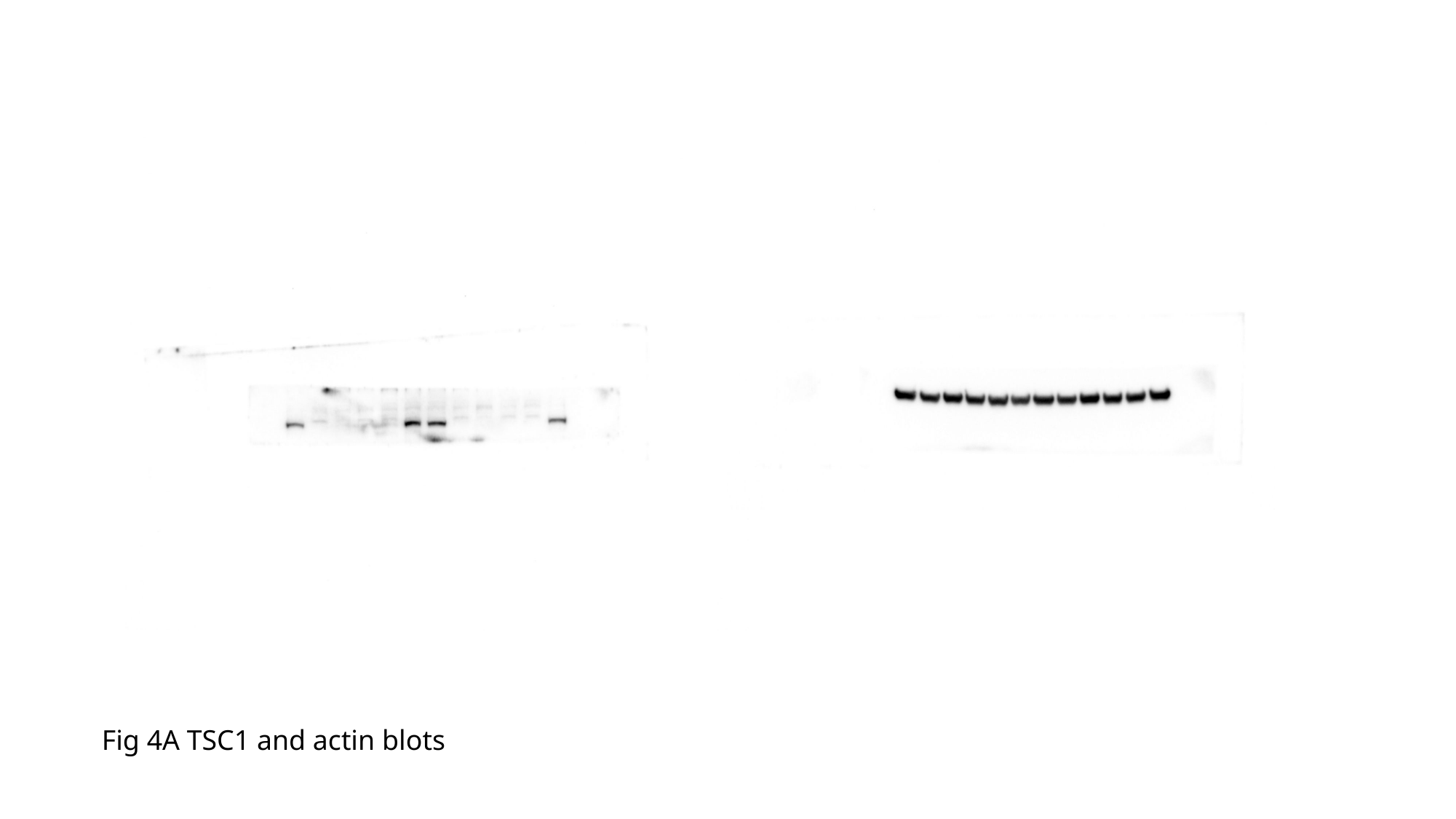

Fig 4A TSC1 and actin blots

## Slide 4
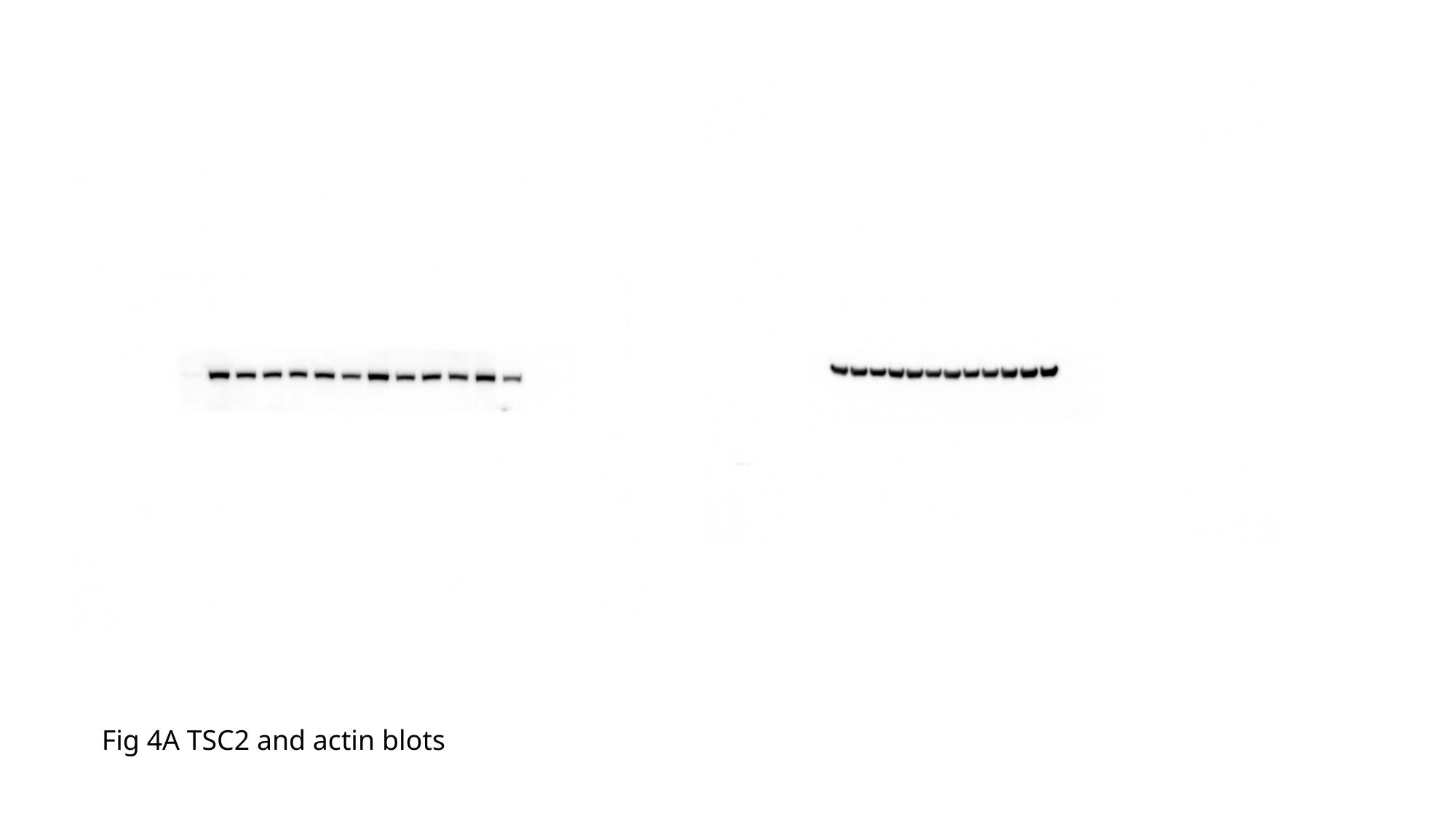

Fig 4A TSC2 and actin blots

## Slide 5
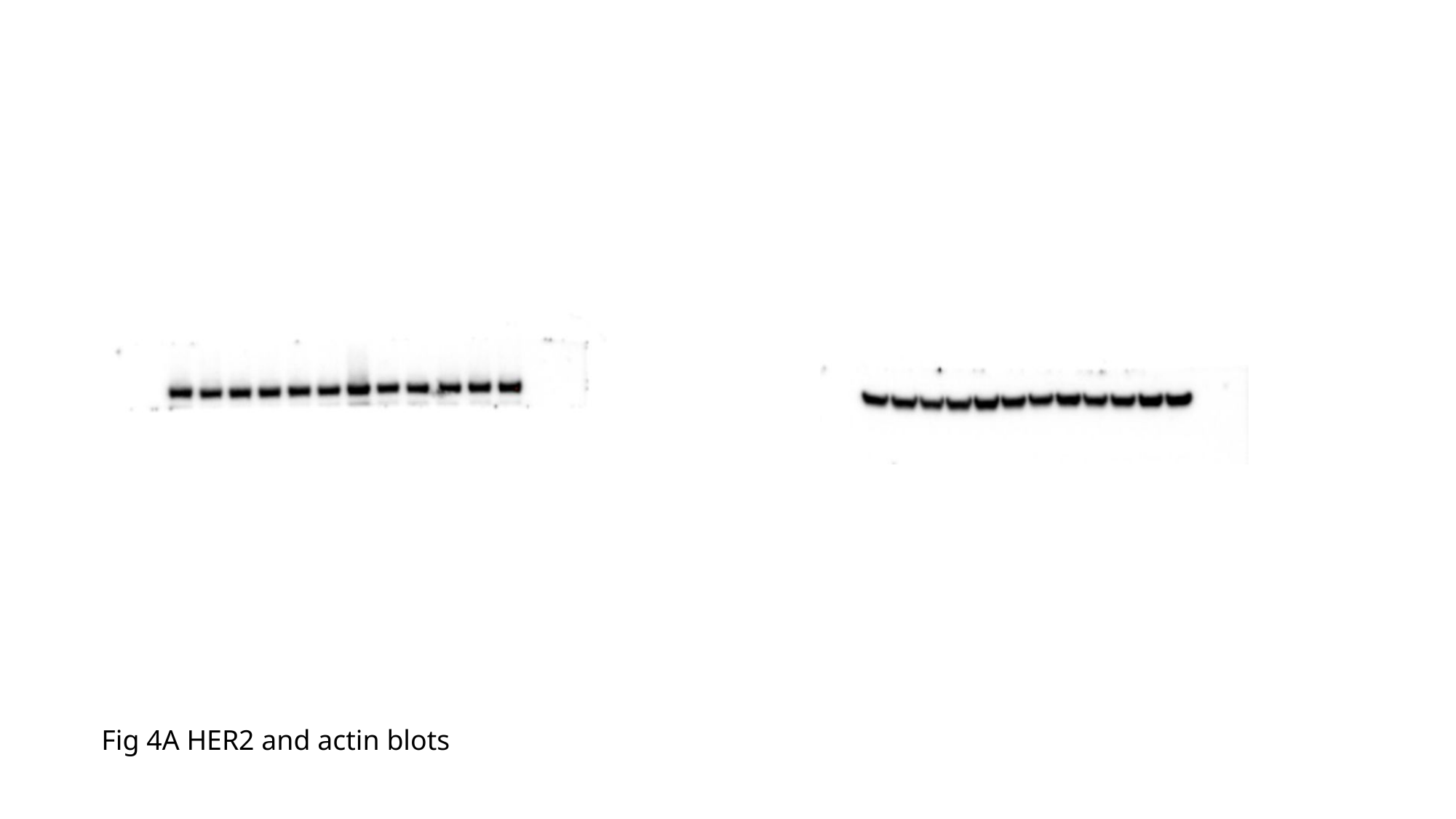

Fig 4A HER2 and actin blots

## Slide 6
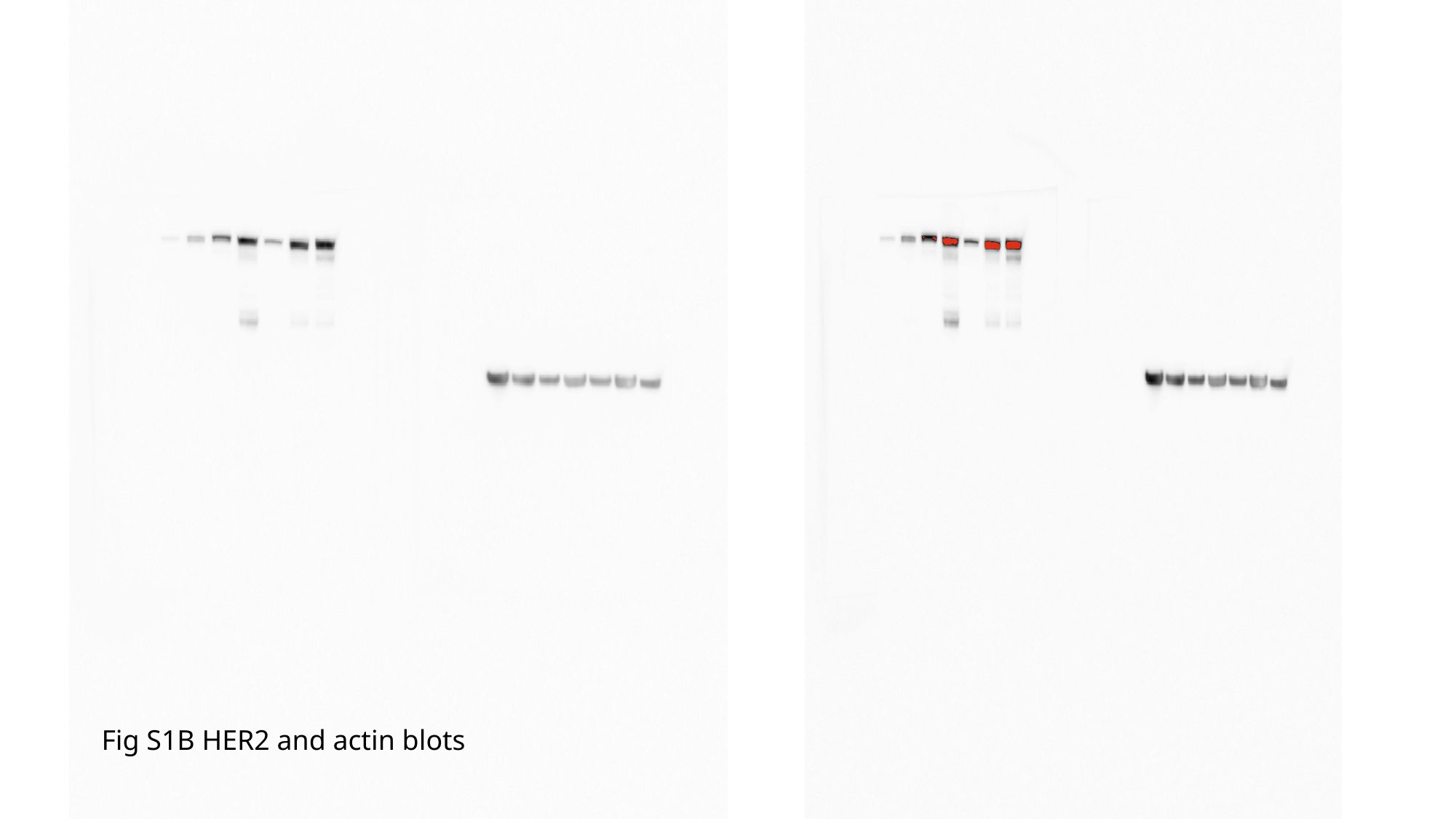

Fig S1B HER2 and actin blots

## Slide 7
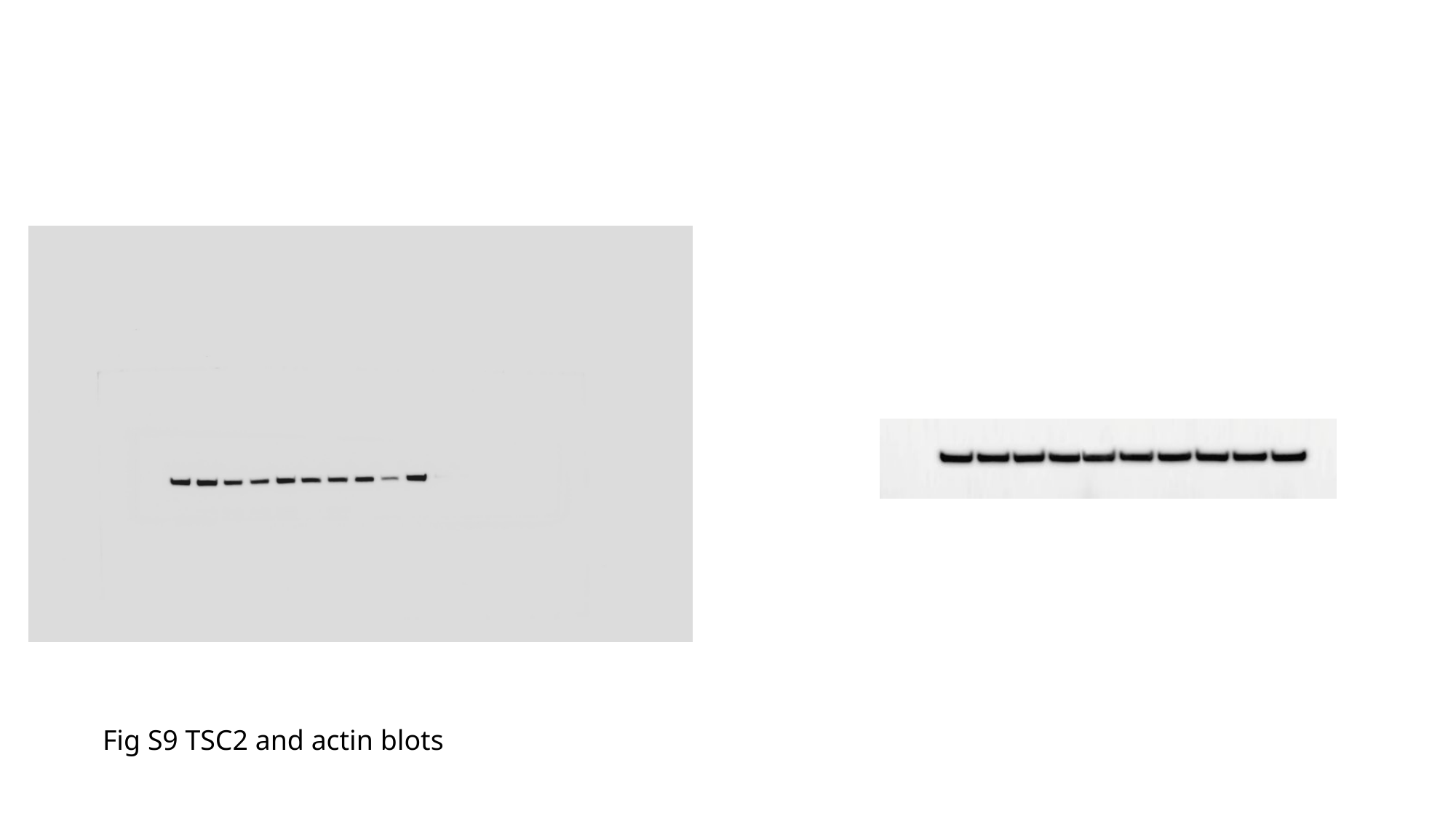

Fig S9 TSC2 and actin blots
